# To Be or Not To Be T4: Evidence of a Complex Evolutionary Pathway of Head Structure and Assembly in Giant *Salmonella* Virus SPN3US

**DOI:** 10.3389/fmicb.2017.02251

**Published:** 2017-11-15

**Authors:** Bazla Ali, Maxim I. Desmond, Sara A. Mallory, Andrea D. Benítez, Larry J. Buckley, Susan T. Weintraub, Michael V. Osier, Lindsay W. Black, Julie A. Thomas

**Affiliations:** ^1^Thomas H. Gosnell School of Life Sciences, Rochester Institute of Technology, Rochester, NY, United States; ^2^Biochemistry, University of Texas Health Science Center at San Antonio, San Antonio, TX, United States; ^3^University of Maryland School of Medicine, Baltimore, MD, United States

**Keywords:** *Salmonella*, myovirus, giant phage, mass spectrometry, prohead protease, CTS (capsid targeting sequence), ejection protein, virion RNA polymerase (vRNAP)

## Abstract

Giant *Salmonella* phage SPN3US has a 240-kb dsDNA genome and a large complex virion composed of many proteins for which the functions of most are undefined. We recently determined that SPN3US shares a core set of genes with related giant phages and sequenced and characterized 18 amber mutants to facilitate its use as a genetic model system. Notably, SPN3US and related giant phages contain a bolus of ejection proteins within their heads, including a multi-subunit virion RNA polymerase (vRNAP), that enter the host cell with the DNA during infection. In this study, we characterized the SPN3US virion using mass spectrometry to gain insight into its head composition and the features that its head shares with those of related giant phages and with T4 phage. SPN3US has only homologs to the T4 proteins critical for prohead shell formation, the portal and major capsid proteins, as well as to the major enzymes essential for head maturation, the prohead protease and large terminase subunit. Eight of ~50 SPN3US head proteins were found to undergo proteolytic processing at a cleavage motif by the prohead protease gp245. Gp245 undergoes auto-cleavage of its *C*-terminus, suggesting this is a conserved activation and/or maturation feature of related phage proteases. Analyses of essential head gene mutants showed that the five subunits of the vRNAP must be assembled for any subunit to be incorporated into the prohead, although the assembled vRNAP must then undergo subsequent major conformational rearrangements in the DNA packed capsid to allow ejection through the ~30 Å diameter tail tube for transcription from the injected DNA. In addition, ejection protein candidate gp243 was found to play a critical role in head assembly. Our analyses of the vRNAP and gp243 mutants highlighted an unexpected dichotomy in giant phage head maturation: while all analyzed giant phages have a homologous protease that processes major capsid and portal proteins, processing of ejection proteins is not always a stable/defining feature. Our identification in SPN3US, and related phages, of a diverged paralog to the prohead protease further hints toward a complicated evolutionary pathway for giant phage head structure and assembly.

## Introduction

In recent years there has been a remarkable realization that standard phage isolation techniques were biased against larger phages and that “giant” or “jumbo” dsDNA tailed phages with genomes >200 kb can be readily isolated from a diversity of environmental samples and locales (Serwer et al., 2004, 2007, 2009; Krylov et al., 2007). The first giant phage genome published was that of *Pseudomonas aeruginosa* phage ϕKZ (280 kb) in 2002 (Mesyanzhinov et al., 2002). Since then even longer phage genomes have been reported, up to 480 kb (*Bacillus megaterium* phageG, Hendrix, 2009). Both ϕKZ and phageG were isolated decades prior to genome sequencing (Donelli, 1968; Krylov et al., 1984) and were considered rare oddities with fascinatingly complex virions but of little general relevance. However, ϕKZ-like phages have been incorporated into therapeutic mixtures of phages for phage therapy of *P. aeruginosa* infections (Krylov et al., 2007, 2012). There has been a surge in interest in phage therapy due to the problems of multi-drug resistant bacteria (Harper and Morales, 2012). Consequently, more than thirty phages related to ϕKZ infecting a range of hosts including *Salmonella* (e.g., SPN3US), *Erwinia amylovora* (e.g., Ea35-70), *Cronobacter sakazakii* (e.g., CR5) and *Vibrio* spp. (e.g., JM-2012) have now been isolated with the goal of developing novel phage-based therapeutics (Lee et al., 2011, 2016; Jang et al., 2013; Meczker et al., 2014; Yagubi et al., 2014; Bhunchoth et al., 2016; Danis-Wlodarczyk et al., 2016). For example, PhiEaH2 is one of two phages in “Erwiphage,” the first marketed bacteriophage-based pesticide against *E. amylovora* in Hungary (Meczker et al., 2014).

There is also great interest in giant phages related to ϕKZ as their long genomes, which range in length from 211 to 316 kb, have many genes without counterparts in other tailed phage taxa. Not surprisingly, these phages have numbers of highly unusual traits relative to other phage types, including virions amongst the most complex of known phages, and atypical mechanisms for replication within the bacterial cell, as highlighted in recent studies (Kraemer James et al., 2012; Ceyssens et al., 2014; Zehr Elena et al., 2014; De Smet et al., 2016; Leskinen et al., 2016; Van den Bossche et al., 2016; Chaikeeratisak et al., 2017). Studies on ϕKZ determined that its virion is unusually large with a T = 27 capsid (Fokine et al., 2005a, 2007) and an odd structure within its head—a large proteinaceous inner body (IB), around which the DNA is tightly wrapped (Krylov et al., 1984). We determined the three-dimensional reconstruction of the ϕKZ IB and estimate it is comprised composed of 15–20 MDa of protein (Wu et al., 2012). That is, essentially the IB represents a large bolus or mass of many different proteins that are ejected into the host cell along with the genome (Wu et al., 2012). As observed for ϕKZ, other relatives of ϕKZ also show “bubblegrams” under the cryo-electron microscope indicative of an IB/ejection protein mass (Thomas et al., 2008; Sokolova et al., 2014).

Mass spectral analyses of phages ϕKZ and relatives *P. aeruginosa* phage EL and *P. chlororaphis* phage 201ϕ2-1 revealed that the virions of these phages are comprised of 60–70 different proteins—twice the number of different proteins found in the virion of the model phage T4 (Fokine et al., 2005a; Thomas et al., 2008, 2012; Lecoutere et al., 2009; Sycheva et al., 2012). The heads alone of phages related to ϕKZ contain ~50 different proteins and the IB/ejection protein bolus is likely the locale for many of these proteins (Thomas et al., 2012). We hypothesize that these structures/proteins are likely multi-functional having roles in assembly, stability and host-takeover (Black and Thomas, 2012; Thomas et al., 2012). Support for the latter is that all related giant phages have a multi-subunit virion RNA polymerase (vRNAP) which is packaged within their heads for ejection into the host cell for production of early gene transcripts (Thomas et al., 2008; Ceyssens et al., 2014). The vRNAP and a second multi-subunit non-virion RNAP (nvRNAP) are a hallmark feature of all giant phages related to ϕKZ and are highly unusual as they are comprised of β and β' subunits which themselves are split into 2–3 subunits. This is very different from the single subunit RNAPs of phages T7 and N4 (Kazmierczak et al., 2002; Sousa and Mukherjee, 2003; Gleghorn et al., 2008) and likely enables transcription of giant phage proteins to be completely, or almost completely, independent of the host transcriptional machinery (Ceyssens et al., 2014; Leskinen et al., 2016).

We determined that head proteins in 201ϕ2-1 and ϕKZ, including the major capsid protein (MCP) and ejection protein candidates, undergo proteolysis by a protease (Thomas et al., 2012; Thomas and Black, 2013). We identified the ϕKZ enzyme, gp175, using bioinformatics analyses and then demonstrated that it cleaved two inner body proteins, making it the first phage protease to be expressed, highly purified and shown to be active *in vitro* (Thomas and Black, 2013). This enzyme is now the type protease for the MEROPs family S80 (Rawlings et al., 2012). Importantly, we identified that ϕKZ gp175 and its homologs in related giant phages, such as SPN3US gp245, share diverged sequence similarity and predicted structural elements with the prohead protease of T4 phage, gp21 (Thomas et al., 2012; Thomas and Black, 2013). Remarkably, the T4 prohead protease has been shown to conserve the structural elements and catalytic residues of Herpesvirus protease (Cheng et al., 2004; Liu and Mushegian, 2004; Rossmann et al., 2007). This finding, in conjunction with shared folds in MCP, portal and large terminase proteins, support a shared ancestor for Herpesviruses and tailed phages (Baker et al., 2005; Fokine et al., 2005b; Rixon and Schmid, 2014).

In T4, proteolytic processing by gp21 is an essential step in head assembly and results in a vast remodeling of the capsid architecture, setting the stage for genome packaging (Black et al., 1994; Miller et al., 2003). Briefly, T4 heads assemble via nucleation of the portal ring on the inner host membrane. Upon this ring a spherical mass of protein assembles, referred to as the core structure, which consists of the scaffold protein, gp22 (576 copies), internal proteins (~1,000 copies), Alt (40 copies), gp67 (341 copies), gp68 (240 copies), and gp21 (100 copies) (Black et al., 1994). All of the T4 internal head proteins associate with the core structure via a capsid-targeting-sequence or CTS (Mullaney and Black, 1996). Upon completion of the core complex the shell then assembles around. The shell is composed of two proteins, the MCP, gp23 (960 copies) and gp24 (55 copies), which is located at the capsid vertices and is a paralog of gp24 (Fokine et al., 2006). At this point, the protease gp21 cleaves the core proteins gp22, gp67, and gp68 into short fragments, as well as *N*-terminal propeptides from gp23, gp24, Alt and the IPs (Showe et al., 1976a,b). Fragments of the scaffold and core proteins, as well as the propeptides of the internal proteins exit the capsid and the capsid shell expands, increasing the internal volume of the capsid by 30–50% (Black and Rao, 2012). The prohead is then released from the host membrane and undergoes the final step of head assembly—DNA packaging where the terminase proteins packages the 170 kb genome into the head to remarkably high density (~500 mg/ml) (Black and Rao, 2012; Black, 2015). The addition of the contractile tail assembled through a separate pathway and two decoration proteins, HOC and SOC, to the exterior of the capsid completes virion assembly.

These steps in T4 assembly, and many other features of this model phage, were elucidated with the aid of an elegant genetic system (Epstein et al., 1963, 2012). Not surprisingly, this system showed that the T4 genes associated with formation and maturation of the head, gps 17, 20, 21, 22, 23, gp24, 67, and 68, are essential (Miller et al., 2003). The proteins packaged within the dsDNA and ejected into the host cell, the IPs and the ADP-ribosyltransferase Alt, are not essential for assembly; however IPI is essential for the inhibition of a Type IV restriction endonuclease in *E. coli* strains containing the GmrSD enzyme gene (Black and Abremski, 1974; Abremski and Black, 1979; Bair et al., 2007; Rifat et al., 2008). Noting the power of such a genetic system and that in all phages related to ϕKZ there is a core set of conserved genes (including genes for major virion proteins and the vRNAPS), we sought to establish *Salmonella* phage SPN3US as a model giant phage genetic system (Thomas et al., 2016). We isolated, sequenced, and characterized 18 amber mutants of SPN3US, identifying 13 essential genes, only two of which, a SbcC and vRNAP β subunit, had been assigned putative functions previously. Illustrating the potential for this system, analyses of a putative neck gene mutant determined that ~50 gene products are present in the mature SPN3US head. Additionally, analyses of the vRNAP β mutant facilitated identification of the previously unidentified *C*-terminal domain of the giant phage vRNAP β′ subunit and suggested a new phenomenon in phage head assembly—the five-subunit vRNAP enzyme complex assembles prior to incorporation into the prohead.

We continue to develop our novel genetic resource with the goal of implementing it to resolve questions regarding giant phage biology. To assist this process, in this study we sought to characterize the wild-type SPN3US virion using mass spectrometry and determine if proteolysis of head proteins occurs, as observed in related phages, and if so, define which proteins are processed and to what degree. In doing so, we aimed to gain insight into the head features and maturation characteristics that are shared in related giant phages and those that are shared between giant phages and T4 phage. Our results show that while SPN3US shares a head maturation protease with diverged sequence similarity to that of T4 and other major head features of T4, there are substantial variations in the proteolysis step in head maturation in SPN3US relative to T4 and also to other giant phages. Unexpectedly, we only identified approximately half the number of different proteins that undergo proteolytic processing in SPN3US vs. the number found in previously analyzed giant phage heads, despite all phages having approximately the same number of overall head proteins and sharing homologous proteins. In addition, our analyses of four SPN3US head gene mutants identified one of the processed head proteins as a novel head ejection protein (gp47), and another (gp243) that is essential for the assembly of the two most abundant, processed head ejection proteins into the prohead. The analyses of two vRNAP subunit mutants provided further evidence to support that the vRNAP is incorporated into the prohead as a multimer.

The identification of a diverged paralog to the prohead protease in SPN3US, and other giant phages, that is truncated and presumably no longer active (i.e., a cryptic protease) suggests a complex evolutionary pathway for the head proteolysis maturation step.

## Materials and methods

### Propagation and purification of phages

Bacterial stocks of *E. coli* and *Salmonella enterica* Typhimurium strains TT9079 and TT6675 and phages SPN3US and T4 were propagated using LB media. SPN3US wild-type and mutant phages were propagated in overlays containing 0.34% agar at 30°C. Phage dilutions were prepared in SM buffer. SPN3US amber mutants were isolated via hydroxylamine mutagenesis as described previously (Thomas et al., 2016).

Amber mutant particles were purified after propagation on the non-permissive host (TT9079) in LB broth supplemented with 1 mM CaCl_2_ and 1 mM MgCl_2_ at 30°C. Typically, an overnight culture was diluted 1:100 and grown to an OD_600_ of 0.3–0.5 at which point phage was added (MOI of 10). Phages were allowed to adsorb for 15 min then cells were pelleted twice at low speed (5,000 rpm, 3 min, RT) and resuspended in fresh media. Infected cells were then incubated with shaking for 3 h 30°C, and then treated with lysozyme (1 mg/ml) at room temperature with gentle shaking for 30 min. Mutant particles were concentrated by differential centrifugation then purified by sequential CsCl step then overnight buoyant density gradient ultracentrifugation as described previously (Thomas et al., 2016).

### SPN3US amber mutant genome sequencing

SPN3US wild-type and mutant phage DNAs were extracted from high titer stocks that had undergone differential centrifugation (titers typically 5 × 10^11^ pfu/ml) and purified using a phage DNA extraction kit (Norgen). Mutant phage genomes underwent genome sequencing at the University of Rochester Genomics Research Center on an Illumina MiSeq machine (2 × 250). Genomes were assembled and SNP reports generated using SeqMan NGen and SeqMan Pro, respectively (DNASTAR). The reference sequence used for alignments was the wild-type SPN3US genome GenBank accession JN641803 (Lee et al., 2011).

### Mass spectrometry

Samples were boiled for 10 min in SDS sample buffer prior to electrophoresis on Criterion XT MOPS 12% SDS-PAGE reducing gels (Bio-Rad) and subsequent protein visualization by staining with Coomassie blue. Gel lanes were divided into slices (10 for the wild-type phage, six for the mutants). Efforts were made to avoid transecting visible stained bands. No replicates of samples were analyzed. After de-staining, proteins in the gel slices were reduced with TCEP [tris(2-carboxyethyl)phosphine hydrochloride] and then alkylated with iodoacetamide before digestion with trypsin (Promega). HPLC-electrospray ionization-tandem mass spectrometry (HPLC-ESI-MS/MS) was accomplished on a Thermo Fisher LTQ Orbitrap Velos Pro mass spectrometer or a Thermo Fisher Orbitrap Fusion Lumos mass spectrometer. Mascot (Matrix Science; London, UK) was used to search the MS files against a locally generated SPN3US protein database that had been concatenated with the SwissProt database (2012_11_170320; version 51.6). Subset searching of the Mascot output by X! Tandem, determination of probabilities of peptide assignments and protein identifications, and cross correlation of the Mascot and X! Tandem identifications were accomplished by Scaffold (Proteome Software). MS data files were either processed individually or the files for an entire gel lane were combined via the “MudPIT” option.

Peptides generated by cleavage by a prohead protease were identified through use of database searching (Mascot and X! Tandem) using an enzyme specificity of “semi-trypsin” followed by visual inspection of the results in Scaffold (Proteome software). This process was necessary because of unknown cleavage specificity of the SPN3US protease. Our previous studies have found variations in protease specificity even between the homologous proteases of ϕKZ and 201ϕ2-1 (Thomas et al., 2010, 2012; Thomas and Black, 2013). The results for identified proteins, numbers of unique peptides, total spectra, and sequence coverage for each experiment were exported from Scaffold with the following quality filters: peptide, 95%; protein, 99.9%; minimum number of peptides, 3. Microsoft Excel was then used to generate spectrum count profiles, as described previously (Thomas et al., 2010). An estimate of the relative abundance of SPN3US virion proteins was calculated by dividing the total number of spectra assigned for each protein (spectral count, SC) in the MudPIT analyses by its molecular mass (SC/ M) as performed for phages 0305ϕ8-36 (Thomas et al., 2007), 201ϕ2-1 (Thomas et al., 2007, 2010), RIO-1 (Hardies et al., 2016), and ϕKZ (Thomas et al., 2012). Our results from other phages have demonstrated that SC/M provides a useful indicator of relative abundance of different virion proteins. That is, proteins with similar SC/M values are typically present in similar relative abundances in the virion. In addition, proteins with SC/M ≤ 1 are likely to be present in only few copies, or even less than one copy, per virion. The copy numbers of several SPN3US virion proteins are known; major capsid protein (gp75), 1560 copies; tail sheath (gp256), 264 copies (Alasdair Steven and Weimin Wu, personal communication); and portal protein (gp81), 12 copies.

**Figure 1:**
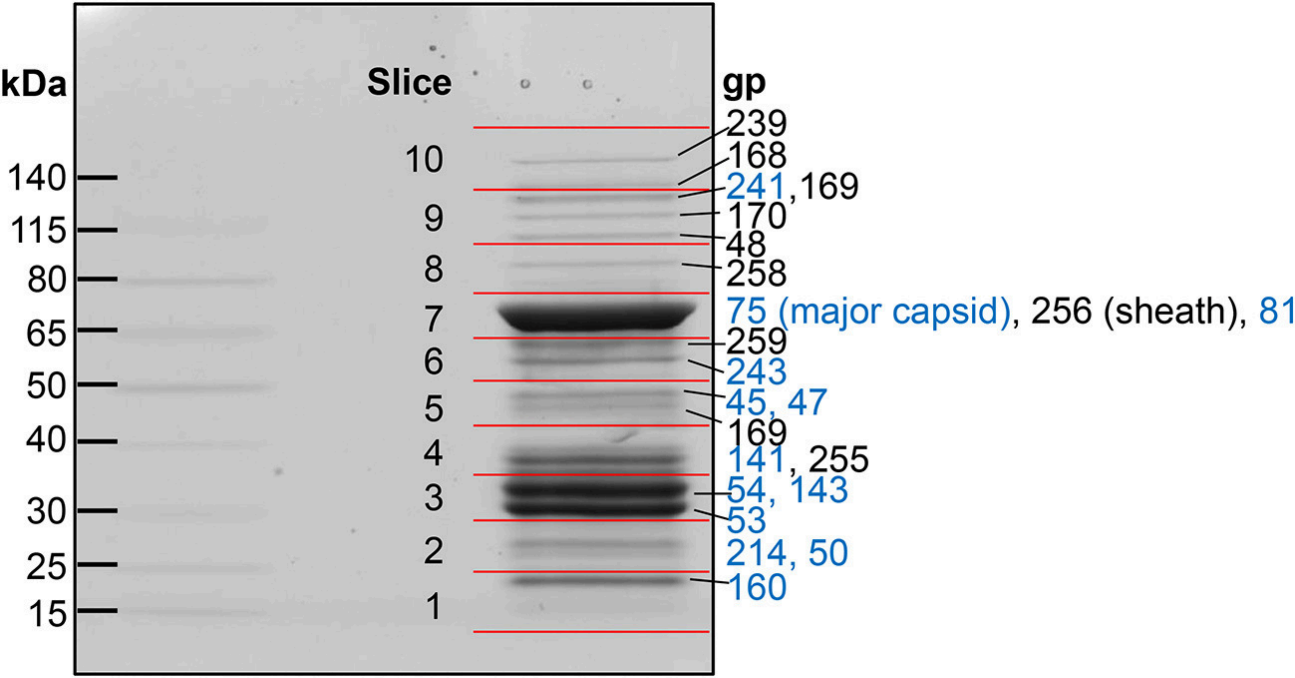
SDS-PAGE gel of purified SPN3US virions. Individual gel slices that underwent mass spectrometric analyses are indicated by red lines. The most abundant SPN3US proteins identified in each gel slice are indicated with gene product (gp) numbers, with the most abundant protein in each slice, or gel band, listed first. Proteins that are components of the head structure are indicated (blue).

There are limitations to estimations of protein abundance using SC/M; therefore we also used densitometry, as conducted previously for the complex myoviruses 0305ϕ8-36 (Thomas et al., 2007) and ϕKZ (Thomas et al., 2012) and the podovirus RIO-1 (Hardies et al., 2016), for additional confirmation of head proteins assigned by SC/molecular mass as highly abundant. For these analyses, as two stoichiometric controls, the major sheath and portal proteins, migrate to the same region of the gel as the broad band of the highly abundant MCP (Figure 1) we used results from SPN3US mutant 64_112(am27) propagated under non-permissive conditions (it has a tailless phenotype) to assess the abundance of high copy number head proteins (Thomas et al., 2016).

### Cloning, expression, and purification of the SPN3US prohead protease

The full-length form of the SPN3US protease (gp245) gene was amplified from SPN3US DNA by PCR using the primers pHS245F (5′-GCGCCATGGAAAACTTGTCACTACGTTATAACTGCGTGGC-3′) and pHS245R (5′- GCGTCTAGATTACCAGCTCCTTACACCCATGCCCATTACC-3′). The gp245 gene was then cloned into the vector pHERD20T (Qiu et al., 2008) using the NcoI and XbaI sites and transformed into *E. coli* DH10B. Codons for a six-histidine tag were subsequently added to the 5' end of gp245 gene via site-directed mutagenesis (SDM) as described in Thomas and Black (2013). PfuUltra Hotstart DNA polymerase (Agilent) was used for the amplifications, and subsequent digestion by DpnI (NEB) was undertaken to remove any remaining template DNA. Plasmid DNA was purified using the QIAprep spin miniprep kit (Qiagen) and the construct verified by DNA sequencing using the pHERD sequencing primers (Qiu et al., 2008). The gp245 construct was propagated in LB broth containing 150 μg/ml of ampicillin until mid-log phase, and protein expression was induced by the addition of arabinose (1% final concentration) for 1 h at 37°C. Cells were pelleted at 6,000 rpm (Sorvall rotor SS34) for 10 min, then resuspended in lysis buffer (20 mM Tris-Cl [pH 7.5], 150 mM NaCl, 1 mM EDTA, and egg white lysozyme [0.3 mg/ml; Sigma]), for ~1 h, 4°C. The lysate was then treated with DNase (40 U/ml; Roche) at 37°C for 20 min, centrifuged (10,000 g, 10 min), and the supernatant was mixed with HisPur nickel resin (Thermo Scientific) overnight at 4°C. Purification of gp245 was performed according to the nickel column manufacturer's instructions at room temperature. Washes were performed in buffer containing 20 mM Tris-Cl (pH 7.5) and 300 mM NaCl with increasing imidazole concentrations (final elution buffer contained 250 mM imidazole). The eluted gp245 was dialyzed against wash buffer containing no imidazole and electrophoresed on a 12% Bis-Tris SDS-PAGE gel (Novagen). The major gel band was excised and digested separately with trypsin and chymotrypsin prior to mass spectrometric analyses as described above.

### Transmission electron microscopy

Purified SPN3US mutant particles were adsorbed to 400 mesh carbon-coated grids and negatively stained with uranyl acetate (1%). Samples were examined at 80.0 kV using a FEI Tecnai T12 transmission electron microscope at the University of Maryland Electron Microscopy Core Imaging Facility.

### Bioinformatics analyses

PSI-BLAST searches were performed on a local implementations of the NCBI BLAST suite (Altschul et al., 1997), as were analyses using the Sequence Analysis and Modeling System (SAM) (Hughey and Krogh, 1996; Karplus et al., 1998) and HHpred (Söding, 2005). Phylogenetic trees were created from alignments created by MUSCLE (Edgar, 2004) using PAUP version 4.0 (Swofford, 2002) with Maximum Likelihood (50% majority rule) and the CDMut model. The majority rule consensus tree was created from 1,000 bootstrap runs.

## Results

### Identification of SPN3US virion proteins by mass spectrometry

Eighty-six different SPN3US proteins were detected by mass spectrometry in CsCl step purified SPN3US, with a protein identification probability of 100% (Figure 1, Supplementary Table 1). This indicates that ~33% of all SPN3US genes encode virion proteins; together, these proteins represent ~46% because there are a number of long virion genes (e.g., gp168 is 5.2 kb). These SPN3US proteins detected in the virion ranged in molecular mass from 7 kDa for gp172 (a protein of unknown function) to 259 kDa for gp239 (the tail tape measure protein) (Table 1, Supplementary Table 1). The proteins were detected across a wide dynamic range of total spectra and sequence coverage, from four total spectra assigned for gp38 (a protein of unknown function) to 1,592 total spectra for gp75 (the MCP), and sequence coverage from 17% for gp49 to 94% for gp141 (Supplementary Table 1). Overall, fifty of the SPN3US proteins identified had sequence coverage of 50% or higher (Supplementary Table 1).

**Table 1:** Abundant and processed virion proteins in purified SPN3US identified by mass spectrometry.

**gp**	**Slice^a^**	**Mass, kDa (Proc. Mass)^b^**	**Total SC^c^**	**SC/M (Proc. M)^d^**	**Essential^e^**	**Paralog family**	**Comment (expected copies per virion)**
75	7	83.9 (70.4)	1,592	18.97 (22.61)			**Major capsid** (1560 copies) Processed ATE-130.
53	3	45.2 (31.5)	662	14.65 (21.02)	Presumed	A	**Head ejection**. Processed AQE-125, AQE-95.
54	3	45.1 (31.9)	643	14.26 (20.16)		A	**Head ejection**. Processed AQE-124.
160	1	18.5	175	9.46		B	Head ejection?
141	4	32.6	306	9.39		B	Head ejection?
256	7	75.7	669	8.84	Presumed		**Tail sheath** (~264 copies)
255	4	32.7	209	6.39	Presumed		**Tail tube** (~264 copies)
**MID-LOW ABUNDANCE PROTEINS**							
243	6	54.6	251	4.60	Yes		Head ejection?
143	3	31.9	118	3.70		B	Head ejection?
45	5	50.3 (48.2)	144	2.86 (2.99)			Head. Processed at ASE-20
47^f^	5	62.8 (50.7)	110	1.75 (2.17)	Yes		Head ejection. Processed AVE-79, expected maturation cleavage ALE-111
50	2	39.4 (25.6)	56	1.42 (2.19)			Head. Processed ATE-127
169	9	149	210	1.41		C	Baseplate/ tail fiber candidate
241	9	159.1	189	1.19	Yes		Head ejection, **vRNAP** β′**N**
168	10	188.1	203	1.08	Yes	C	Baseplate/tail fiber candidate
218	2	25.2	27	1.07	Yes		Head ejection, **vRNAP** β**C**
42	5	49.3	52	1.05			Head ejection, **vRNAP** β′**M**
170	9	135.4	135	1.00		C	Baseplate/ tail fiber candidate
245	2	30.7 (23.4)	25	0.81(1.06)	Presumed		Head **Prohead protease**, Processed AQE-203,
81	7	100.2 (72.3)	93	0.93 (1.29)			Head **Portal (12 copies)** Processed ATE-161, expected AQE-254
240	6	59.6	33	0.55	Yes		Head ejection, **vRNAP** β′**N**
244	2	27	12	0.44	Yes		Head ejection, **vRNAP** β′**C**

*All highly abundant proteins are shown. Several mid- and low abundance proteins, such as the portal, prohead protease, and vRNAP subunits to highlight the range of abundances detected in the virion. All identified SPN3US proteins are listed in Supplementary Table 1. The slice in the SDS-PAGE gel (Figure 1) in which the spectral count for each protein peaked is provided. Relative abundance was determined from spectral counts adjusted for molecular mass (SC/M). Proteins identified as part of the head are indicated*.

a *The slice in the SDS-PAGE gel (Figure 1) in which the spectral count for each protein peaked*.

b *“Proc. M” indicates molecular mass after processing by the prohead protease, gp245*.

c *“SC” represents the total number of spectral counts for each protein, as determined by the mass spectrometric MudPIT analyses*.

d *“SC/M” indicates total spectral count adjusted by molecular mass. Numbers provided in parentheses are the total spectral count adjusted by the processed molecular mass*.

e *“Essential”—indicates a protein encoded by a gene determined to be essential by the isolation and sequencing of an amber mutant of SPN3US*.

f *Mass spectral analyses re-assigned the start site of the gp47 gene to at nucleotide position 44887 in JN641803.1 which has additional four codons to the predicted start site. Processing sites of the prohead protease in gp47 are for the new peptide co-ordinates (see text)*.

Spectral counting (SC) is an accepted semi-quantitative approach for estimation of relative protein abundance (Zybailov et al., 2005) and was used for this purpose for the SPN3US virion proteins. For each protein we calculated a SC/M value by dividing the total number of spectra assigned to each protein by its predicted molecular mass (Table 1, Supplementary Table 1). Several proteins were found to have undergone proteolytic processing (see below), and in these instances, an accordingly adjusted molecular mass was used to estimate relative abundance. After these adjustments, the protein in the virion with the highest SC/M value (22.6) was gp75, consistent with the expectation that the major capsid protein would be the most abundant protein in the virion. There are 1,560 copies of gp75 per virion based on the SPN3US capsid having the same triangulation number (T = 27) as ϕKZ (Alasdair Steven, Weimin Wu, personal communication). The SC/M values for gp53 and gp54 were 21.0 and 20.2, respectively, more than 2-fold the SC/M values obtained for the tail sheath and tube proteins. Two other SPN3US proteins, gp141 and gp160, had slightly higher SC/M values than the tail sheath SC/M value. The SPN3US tail sheath and tube proteins are expected to be present in ~260 copies per particle based on the following considerations: 1. The SPN3US tail is of similar length as the ϕKZ tail, 2. SPN3US has sheath and tube proteins that are homologous to those of ϕKZ, 3. The ϕKZ tail is known to have 264 copies of each protein in its tail (Fokine et al., 2007).

The SC/M values for gps53 and 54 indicated that their respective copy numbers in the virion were higher than those for the tail sheath and tube proteins. This was unexpected because these proteins are incorporated inside the capsid shell with the DNA. However, the high abundance of gp53 and gp54 is consistent with the SDS-PAGE profile of this phage. To further explore the relative abundances of these two proteins, along with gp141 and gp160, we conducted densitometry analyses of a recently identified tailless mutant [*64_112*(am27); not shown]. These analyses supported our assignment of all four proteins as high abundance virion proteins and we conservatively estimate there are >600 copies each of gps 53 and 54 per capsid and >300 copies each of gps 160 and 141 per capsid.

Many of the SPN3US virion proteins are expected to be present in only a few copies per virion based on the expectation that their SC/molecular mass value is similar to that of gp81, the portal protein which is present in a dodecametric ring situated at a specialized vertex where the head joins the tail in all tailed phages. While some of the SPN3US low abundance proteins may not be true virion or assembly proteins, we expect that many are, based on the fact that the vRNAP subunits (gps 42, 218, 240, 241, and 244, Table 1) have low SC/molecular mass values, but are known to be essential and are packaged into the phage head. Similarly the low abundance of the prohead protease, gp245, is consistent with the expectation that only a few copies are present in the mature capsid, based on the estimation that the T4 protease is only present in about three copies per capsid (Black et al., 1994). The lack of detection of known non-virion proteins [such as the terminase protein (gp260) and DNA polymerase subunits (gp18 and gp44)] in the SPN3US virion provides further support that the low abundance proteins are true virion proteins.

### Identification of three paralog families in the SPN3US virion

Twenty-five of the SPN3US proteins identified by mass spectrometry have homologs to other SPN3US virion proteins, as determined by PSI-BLAST. We categorized these paralogs into three families—Paralog families A, B and C (see examples in Table 1; all are included in Supplementary Table 1). Of these families, the three members of Paralog family C, the low abundance proteins gps 168, 169, and 170 (molecular masses of 188, 149, and 135 kDa, respectively), are the only ones for which a putative function has been deduced. These proteins are most likely related to the baseplate or fibers as they have similarity to ϕKZ gp131 (Sycheva et al., 2012). Supporting this expectation is the fact that these proteins were not identified in a tailless mutant [*64_112*(am27)] (Thomas et al., 2016). No putative function has been discovered, as yet, for Paralog families A and B.

SPN3US Paralog family A has two members, the highly abundant gp53 and gp54, both having similarity to pfam12699. Notably, the number of proteins with similarity to pfam12699 varies in different giant phage. In ϕKZ, for example, there are five homologs: the inner head proteins gps 93, 94, 95, 162, and 163 (Thomas et al., 2012) which are expected to be part of the IB and, therefore, excellent candidates for head ejection proteins. Interestingly, while ϕKZ gps 93, 95, and 162 are expected to be high abundance (>100 copies each per virion), their estimated copy numbers are much lower than our estimates for SPN3US gp53 and gp54, implying that there is significant variation in the amounts of paralogs/homologs in different phages.

SPN3US Paralog family B is highly unusual because of the large number of members (20, gps83, 138–154, 161, and 237), all of which are expected to be head proteins since they were detected in the tailless mutant (Thomas et al., 2016). In addition, we believe that gp122 may be an additional member of this family based on PSI-BLAST searches; however, since gp122 was not detected in the mass spectral analysis of the wild-type phage, this assignment is speculative at this point. Unlike the other two SPN3US paralog families in which the members are present in similar relative abundances, the members of Paralog family B have relative abundances that range from low for most members, to middle (gp143) to high abundance (gps 141 and 160) (Table 1, Supplementary Table 1). There are no counterparts to any of the SPN3US paralog families in T4, although T4 has its own paralogs. As noted above, the T4 shell is composed of the MCP, gp23 and its paralog gp24, which forms the pentameric vertices of the capsid (Fokine et al., 2006).

### Proteolytic processing of SPN3US head proteins

**Figure 2:**
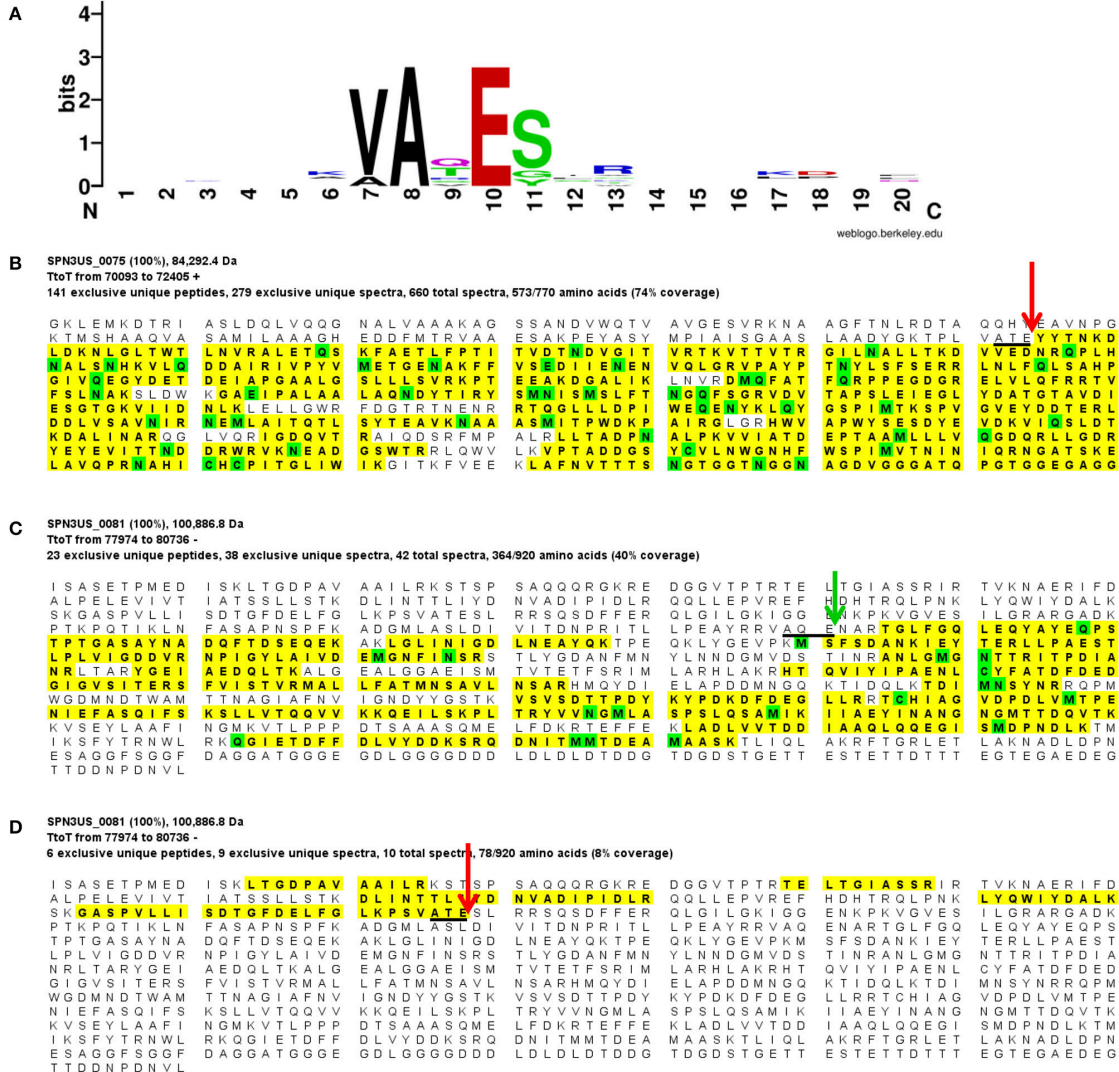
Proteolytic processing of SPN3US head proteins. **(A)** Sequence logo representing 9 cleavage sites confirmed by the identification of semi-tryptic peptides, **(B)** Peptide coverage of the major capsid protein, gp75 **(C)** Peptide coverage of the mature form of the portal protein, gp81 identified in slice 7 (Figure 1), and **(D)** peptide coverage of the cleaved N-terminal region of gp81 in slice 1 (Figure 1). Red arrow indicates maturation cleavage site, green arrow indicates inferred maturation cleavage site.

**Figure 3:**
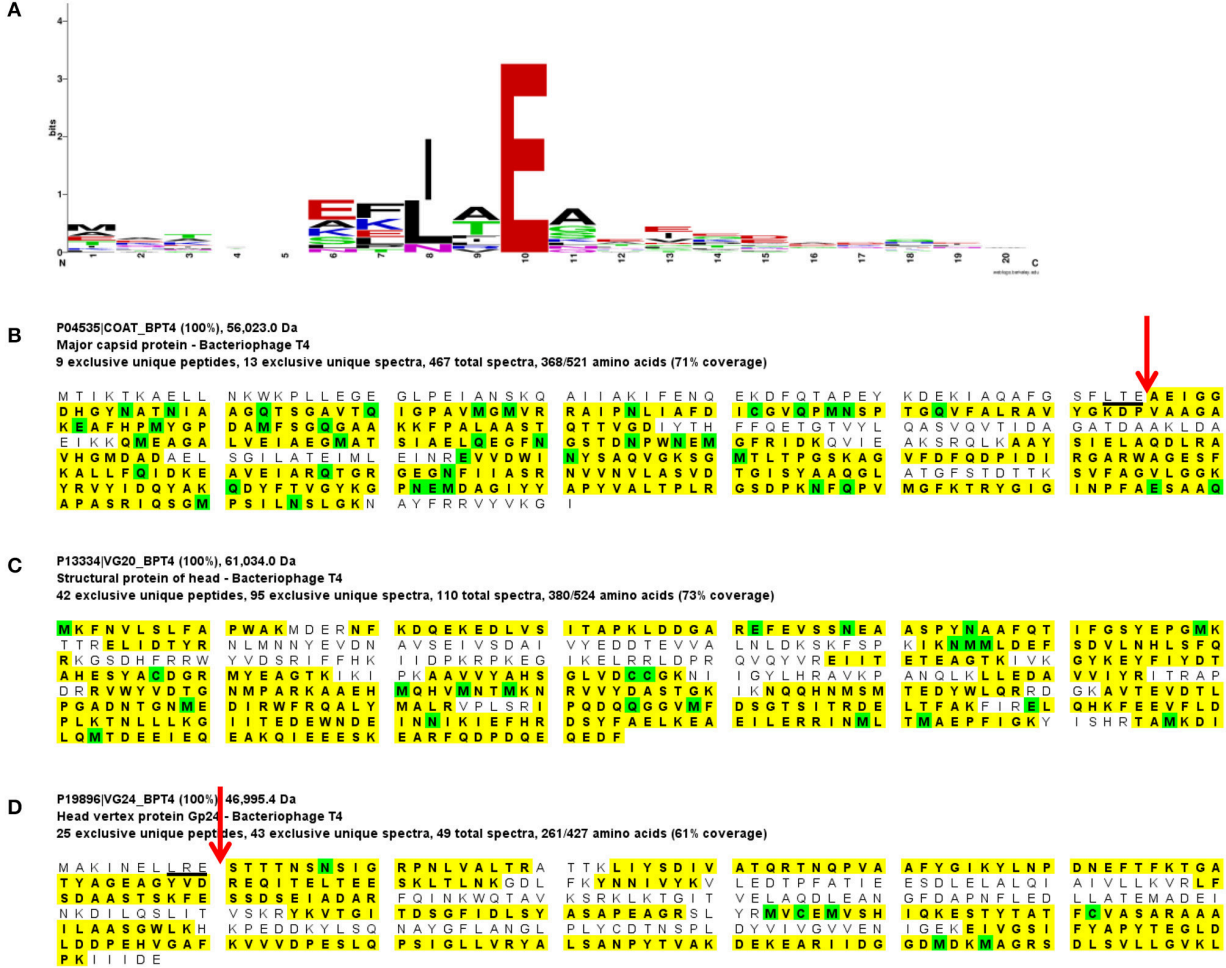
Proteolytic processing of T4 head proteins. **(A)** Sequence logo representing 13 cleavage sites in 9 head proteins confirmed by *N*-terminal sequencing (Black et al., 1994) and/or semi-tryptic peptides (this study). **(B)** Peptide coverage of the major capsid protein, gp23, **(C)** Peptide coverage of the portal protein, gp20, which is not processed, and **(D)**. Peptide coverage of the capsid vertex protein, gp24. Red arrow indicates maturation cleavage site.

Our analyses determined that eight SPN3US head proteins, gps 45, 47, 50, 53, 54, 75, 81, and 245, undergo proteolytic processing by the prohead protease gp245. Processing of these proteins was observed in the mass spectral analyses of the wild-type phage (except for gp245, see below), and additionally in mutant phage samples, both in this work and in our previous analyses (Hardies et al., 2016). In all instances, processing occurred *C*-terminal to a glutamic acid, at the motif A-X-E, where X is any amino acid (Figure 2). This is analogous to the cleavage motifs of the proteases of giant phages ϕKZ and 201ϕ2-1 (Thomas et al., 2010, 2012), as well of that of T4 phage (Black et al., 1994) (see Figure 3), all of which cleave *C*-terminal to a glutamic acid. The SPN3US protease processing sites were identified by detection of semi-tryptic peptides (e.g., gp75 and gp81 in Figure 2). Of the processed SPN3US proteins, only three have known functions: gp75, the MCP; gp81, the portal protein; and gp245, the prohead protease. The precursor form of gp75 has a molecular mass of 83.9 kDa, but after the removal of 130 residues by processing, the predicted molecular mass of this mature form is 70.4 kDa which is consistent with its SDS-PAGE gel migration (Figure 1). This *N*-terminal propeptide of SPN3US gp75 is twice the length of the 65 residue propeptide of the MCP gp23 of phage T4 (Figure 3).

The SPN3US portal gp81 (Figure 2C) has an immature form with a predicted molecular mass of 101 kDa from which a long *N*-terminal propeptide is removed (Figure 2D). This propeptide region of gp81 is at least 161 residues based on the identification of a small semi-tryptic fragment that was detected in the lowest molecular mass gel slice. However, based on the gel migration of the mature fragment of gp81 (where the peak of the spectral counts for gp81 were detected; slice 7, Figure 2) and its peptide coverage, we expect gp81 is also cleaved *C*-terminal to the sequence AQE-254 (Figure 2C). Processing at this site in gp81 would produce a mature form with a molecular mass of 72.3 kDa which is consistent with its gel migration. Despite the absence of similarity at the sequence level between the SPN3US portal and that of T4, as determined by PSI-BLAST, I-TASSER (Roy et al., 2010) structure prediction of the mature polypeptide of gp81 identified its most similar structural homolog as T4 gp20 (Sun et al., 2015) and predicted domains consistent with the crown, wing, stem and clip observed in the portal proteins of several other tailed phages (Orlova et al., 2003; Lhuillier et al., 2009). That is, it is the long propeptide of gp81 that markedly delineates the SPN3US portal from that of T4. Supporting this we confirmed for T4 gp20 for the first time biochemically by mass spectrometry that it is not processed (Figure 3). The function of the long propeptide of SPN3US gp81 can only be speculated upon, but we presume it has a role in head assembly, possibly helping to anchor ejection and/or core proteins during assembly.

Four other SPN3US head proteins have *N*-terminal propeptides longer than 100 residues, including gps 50, 53, and 54, with propeptides of 127, 125, and 124 residues, respectively. These propeptides are considerably longer than the 10–20-residue *N*-terminal propeptides of T4 head proteins, such as gp24 (Figure 3) and its IP proteins (Supplementary Figure 1), referred to as capsid targeting sequences (CTS) for their role in ensuring a protein is incorporated into the prohead (Mullaney and Black, 1996). Of the SPN3US head proteins that undergo *N*-terminal processing, only gp45 has a propeptide of comparable length (20 residues) to the T4 CTS sequences.

**Figure 4:**
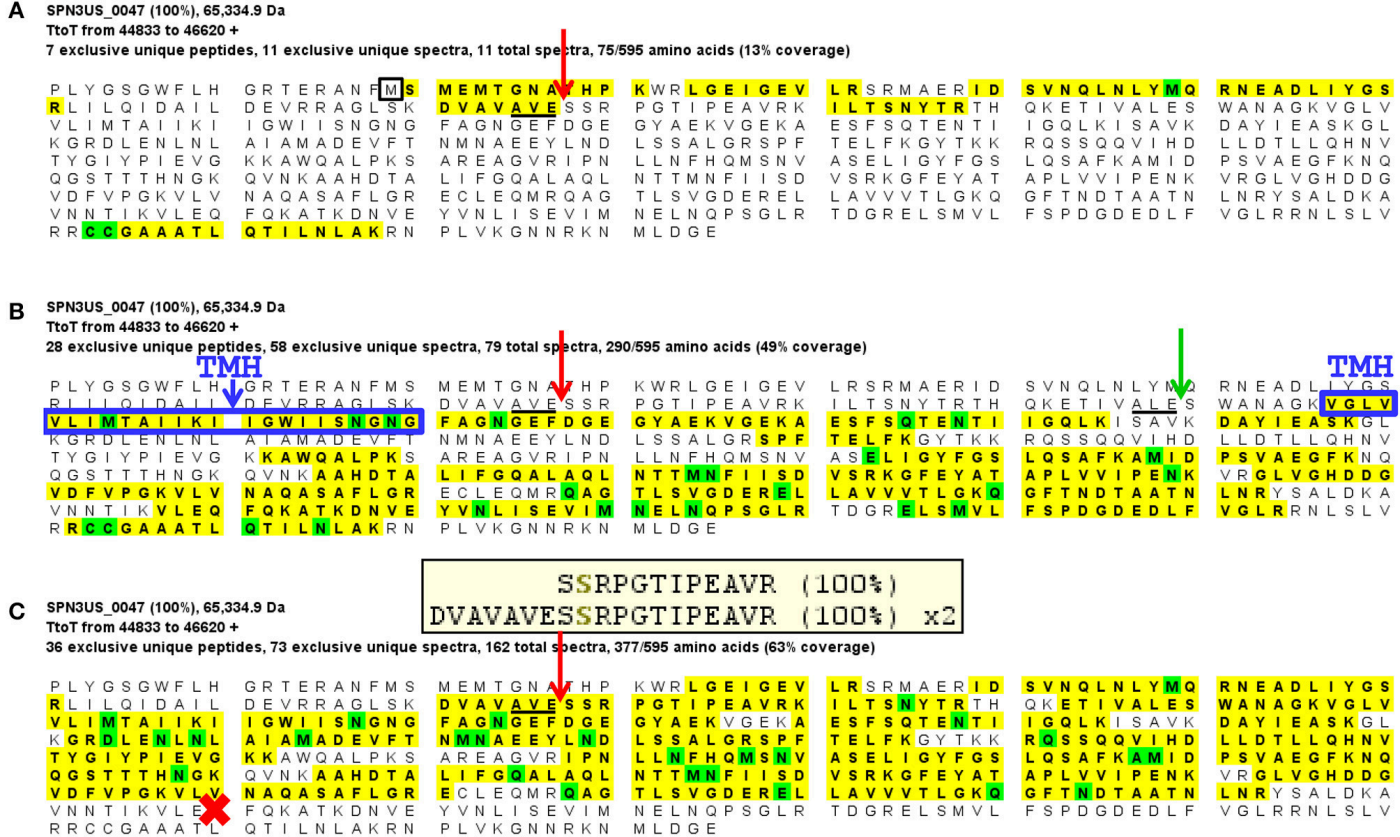
Mass spectral identification of SPN3US ejection protein candidate, gp47. **(A)** Peptide coverage of the precursor propeptide of gp47 identified in the wild-type phage detected in a low molecular mass SDS-PAGE gel slice (slice 1, Figure 1). Red arrow indicates the prohead protease cleavage site (AVE-79) identified via a semi-tryptic peptide; black square indicates newly identified start methionine, **(B)** Peptide coverage of the mature polypeptide of gp47 identified in the wild-type phage detected in a higher molecular mass SDS-PAGE gel slice (slice 5, Figure 1). Green arrow indicates expected protease maturation cleavage site at ALE-111; blue box indicates predicted transmembrane helix (TMH), and **(C)** Peptide coverage of gp47 in the mutant *47*(am1) propagated under non-permissive conditions causing gp47 to be truncated at Q482 (red cross). Note the prohead protease cleavage site from **(A)** is only partially cleaved in this mutant as demonstrated by the identification of both a semi-tryptic peptide that illustrates processing and a tryptic peptide that illustrates no processing at the cleavage motif, 100% refers to the peptide identification probability.

Gp47 also has a long *N*-terminal propeptide, and while it is processed at residue 79 (new co-ordinate, see below), because there was no MS sequence coverage until residue 119 it is more likely that its maturation cleavage is after the sequence which adheres to the cleavage motif: ALE-111 (new co-ordinate) (Figure 4). Our analyses also confirmed a new start site for gp47 as three amino acids were detected by mass spectrometry in the tryptic peptide SME*M*TGNAPHTK which are upstream of its predicted start methionine (underlined) of gp47 (Figure 4A). The codon immediately upstream of this peptide is a methionine leading us to conclude the start site of the gp47 gene is at nucleotide position 44887 in JN641803.1 rather than the predicted nucleotide position 44899. The *N*-terminal methionine of gp47 is likely cleaved by the host methionine aminopeptidase (Wingfield et al., 1989; Movva et al., 1990). The new start methionine codon for gp47 has a credible upstream ribosomal binding site that results in a small overlap of this region with the 3' end of the gp46 gene.

Several of the long propeptides of SPN3US proteins have additional sequences that are consistent with the protease processing motif and may also be cleaved by protease. For instance, the *N*-terminal 15 residues (MANFVKSKLARESVE) of the processed paralogs gp53 and gp54 are identical and contain a sequence (AXE-12) consistent with the known cleavage motif for SPN3US and a sequence (SXE-15) consistent with the ϕKZ protease cleavage motif. We infer that gp53 and gp54 are processed at one or both of these sites, because the ϕKZ homolog, gp93, is processed at SLE-13 (Thomas and Black, 2013). The advantage of additional cleavage sites within propeptide regions would be to produce smaller fragments more easily cleared from the capsid during maturation. That the N-termini of gp53 and gp54 are identical is notable as overall the proteins have diverged extensively from another at the sequence level, as evidenced by their having only 34% identity by BlastP. The sequence conservation at the *N*-termini of gp53 and 54 is reminiscent of the sequence conservation in several of the T4 CTSs and suggests they have a conserved/important role in assembly/maturation.

It is feasible that there may be a small number of additional low abundance proteins in SPN3US that undergo proteolytic processing that were not identified, as peptide coverage is naturally lower in lower abundance proteins. Peptide detection is also impacted by the cleavage specificity of trypsin and resultant peptide length. However, we believe the number of any additional processed proteins to be small based on our examination of eight mass spectrometric SPN3US samples and the identification of intact N- and/or C-termini in many proteins. Identification of any potential additional processed proteins would likely require biochemical assays of recombinant proteins and/or a mass spectrometric analyses of a sample that underwent electrophoresis through a much longer SDS-PAGE gel and consequent division into many slices to enable clear identification of any aberrant gel migration relative to that expected based on the predicted molecular mass.

### Auto-proteolytic processing of the SPN3US prohead protease

**Figure 5:**
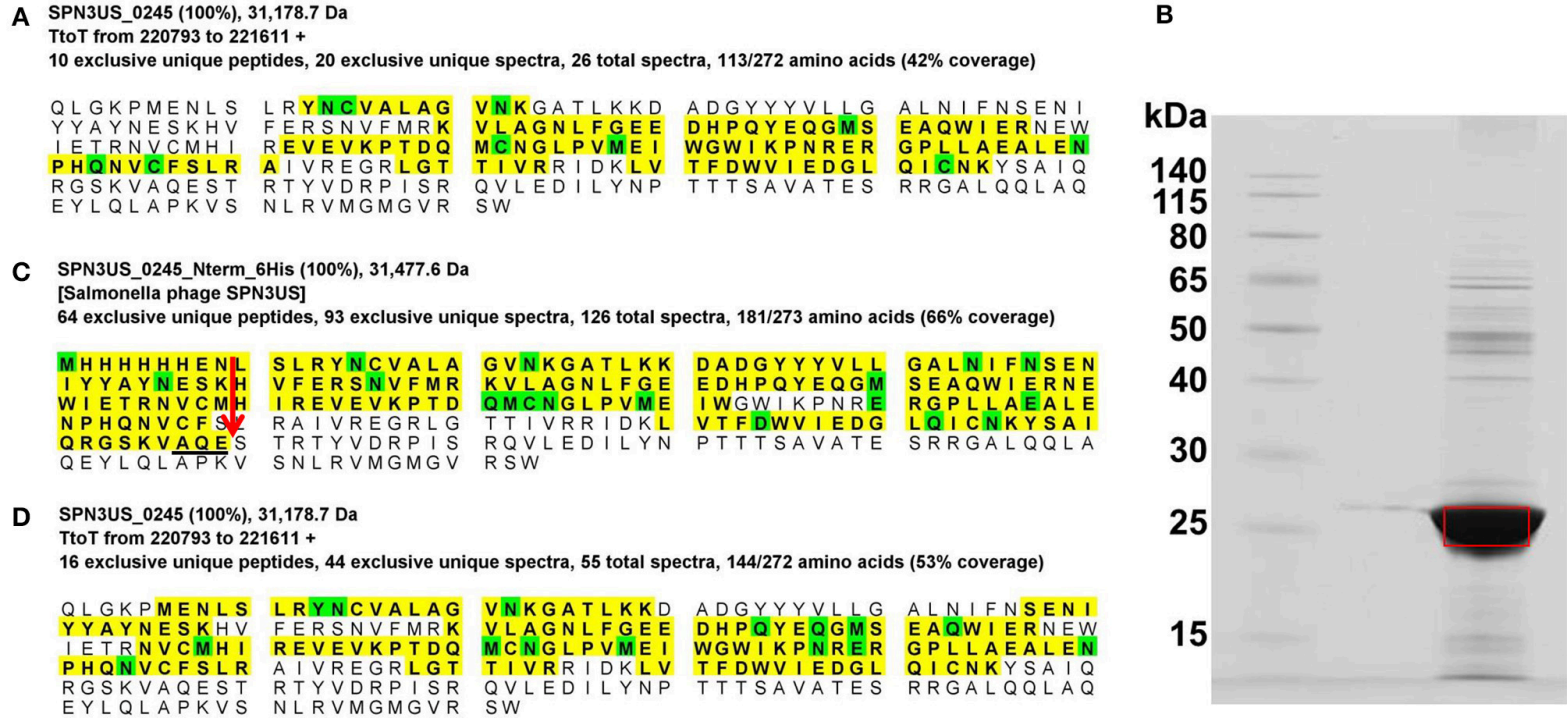
Auto-proteolytic processing of the SPN3US prohead protease, gp245. **(A)** Peptide coverage of gp245 in purified virions (slice 2, Figure 1), **(B)** SDS-PAGE gel of gp245 expressed from the full length gene with an *N*-terminal 6-histidine tag, **(C)** Peptide coverage of recombinant gp245 with an *N*-terminal 6-Histidine tag, and **(D)** Peptide coverage of gp245 in purified virions of *218* that had been propagated under non-permissive conditions. Red arrow indicates the prohead protease cleavage site (AQE-203) identified by a semi-tryptic peptide.

The SPN3US prohead protease, gp245 (263 residues), was identified in the wild-type phage particle as a low abundance protein (Table 1). The mass spectral sequence coverage for gp245 ended at residue K-190 (Figure 5) which is *N*-terminal to three possible cleavage motifs [AQE-246, ATE-234, and AQE-203], leading us to suspect that gp245 underwent C-terminal autocleavage, as we had observed for the ϕKZ protease (Thomas and Black, 2013). To test this hypothesis, we cloned the full-length gp245 gene with additional codons for an *N*-terminal 6-histidine tag in the expression vector pHERD20T and purified the recombinant enzyme on a nickel column. The migration of 6His-gp245 in SDS-PAGE (Figure 5B) was consistent with a lower molecular mass than the predicted 30.7 kDa of the full-length enzyme. This protein band was excised from the gel, digested with chymotrypsin and trypsin and analyzed by mass spectrometry. The resulting peptide coverage obtained for 6His-gp245 identified the *C*-terminus of gp245 (Figure 5C) at glutamate residue 203, confirming its autoprocessing. Cleavage at glutamate 203 produces a mature species with a molecular mass of 23.4 kDa, however it is feasible that one or both of the downstream glutamate residues (residues 234 or 246) are also cleaved. Conversely, we were able to deduce the *N*-terminus of gp245 does not undergo auto-cleavage based on the sequence coverage of gp245 in several samples [e.g., *218*(am101), Figure 5D] and the fact that there are no sequences that contain the AXE-cleavage motif in this region.

We were unable to detect the T4 prohead protease gp21 in T4 heads by mass spectrometry, although it was estimated to be present in the capsid at approximately three copies (Black et al., 1994). The lack of identification may have been a consequence of variations in virus propagation, purification, or there may be a biological cause, such as if in our strain of T4 the protease is efficiently and completely cleared from the prohead. (When gp21 is first packaged into the head, it is estimated to be present in ~100 copies, Black et al., 1994). However as a consequence of not detecting gp21, we were unable to determine if either or both of its suggested autocleavages [*C*-terminal (Keller and Bickle, 1986) or *N*-terminal (Fokine and Rossmann, 2016)] occur. Interestingly, in these studies we made a new observation regarding proteolytic processing of the T4 ejection protein Alt finding it to undergo both *N*-terminal and *C*-processing by gp21 to produce a mature protein of 68.2 kDa (see Supplementary Figure 1C). In addition to the known six residues removed from the *N*-terminus of Alt (by processing at the motif ITE-6), 63 residues are also removed from the *C*-terminus (by processing at the motif LTE-619). This new finding explains the previously noted aberrant SDS-PAGE migration of Alt (faster than expected) and raises questions as to the role(s) of the two propeptides flanking Alt—are they targeting, tethering, and/or controlling its activity? The processing of both termini of Alt highlights the need for future work to clarify the processing status of the T4 protease.

### Characterization of SPN3US essential head protein mutants

**Figure 6:**
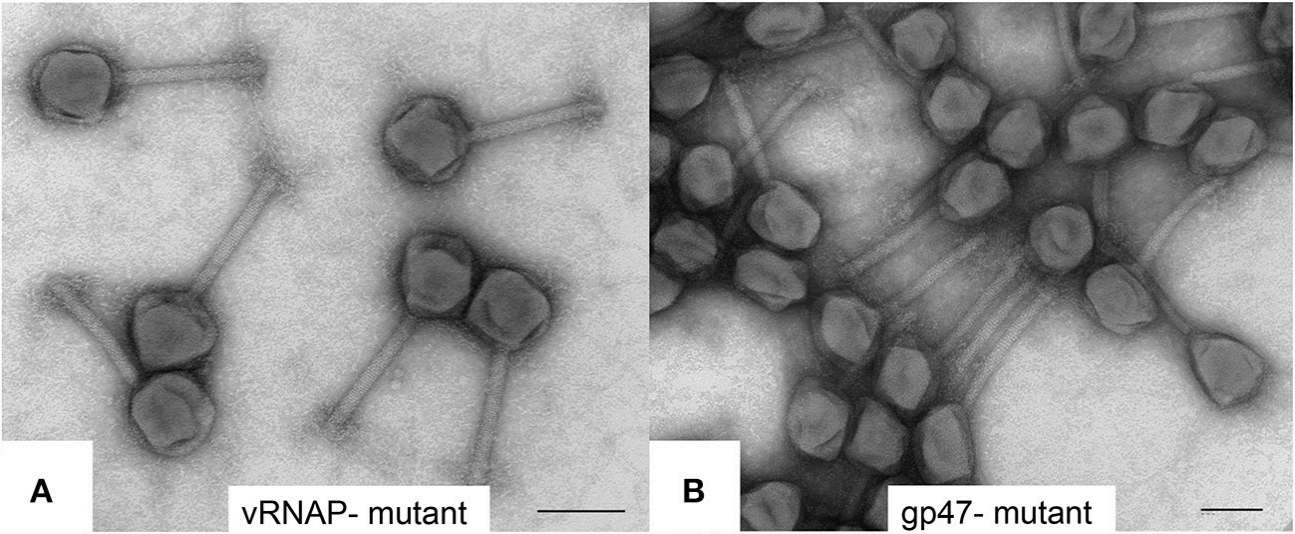
Transmission electron microscopy of negatively stained SPN3US amber mutants **(A)** vRNAP minus mutant [*218*(am101)] and **(B)** ejection protein gp47 minus mutant [*47*(am1)] after propagation on nonpermissive *S. enterica* serovar Typhimurium (strain TT9079). Space bar represents 100 nm.

To further characterize SPN3US head composition and assembly, we examined the effect on the virion when several essential head proteins (gps 47, 218, 243, and 244, Table 1) were knocked out. To do this we utilized amber mutant phages *47*(am1) (Thomas et al., 2016) and newly isolated mutants *218*(am101), *243*(am114), *244*(am84). The genome of each mutant was sequenced and an individual amber mutation in a single gene was identified in all, allowing us to conclude that each gene and its product are essential (Table 2, Supplementary Table 2). Putative functions could only be assigned to the products of two mutated genes, gp218 and gp244, which represent the *C*-terminal subunits of the vRNAP β and β′, respectively (Thomas et al., 2016). These vRNAP subunits were knocked out by propagating the respective mutant under non-permissive conditions and the resulting particles purified via consecutive CsCl step and buoyant density ultracentrifugation gradients. For both mutants, seemingly intact virions were formed, as judged by the gross morphological features (e.g., DNA-filled head, sheath uncontracted, baseplate/tail fibers) and dimensions typical of the wild-type phage (e.g., Figure 6A). However, the purified *218*(am101) and *244*(am84) particles were not viable when propagated on either the permissive or non-permissive host. Mass spectrometric analysis of each mutant proteome revealed that neither mutant had the full complement of virion proteins that were identified in the wild-type phage (Table 2, Supplementary Table 3). Notably, none of the five vRNAP subunits (gps 241, 218, 240, 42, and 244) were identified when either gp218 or gp244 were knocked out, providing further support for our recent identification of gp244 as the fifth subunit of this enzyme and our hypothesis that the vRNAP is assembled as a multimer prior to its incorporation into the prohead (Thomas et al., 2016).

**Table 2:** SPN3US virion proteins not identified in mass spectrometric analyses of amber mutants grown under non-permissive conditions.

**Mutant**	**Mutated gene product, gp**	**vRNAP subunits not identified, gp**	**Other SPN3US virion proteins not identified, gp**	**SPN3US proteins identified, that were not identified as virion proteins in the wild-type phage, gp**
*47*(am1)	47		38 (unknown function) 157 (head protein of unknown function)	100 (thymidylate kinase) 122 (paralog Family B candidate) 158 (unknown function)
*218*(am101)	218	42 (β′M) 218 (βC) 240 (β′N) 241 (βN) 244 (β′C)	38 (unknown function) 41 (unknown function) 98 (unknown function) 157 (head protein of unknown function)	33 (unknown function) 100 (thymidylate kinase) 158 (unknown function)
*244*(am84)	244	42 (β′M) 218 (βC) 240 (β′N) 241 (βN) 244 (β′C)	38 (unknown function) 41 (unknown function) 98 (unknown function) 157 (head protein of unknown function)	28 (unknown function)

**Figure 7:**
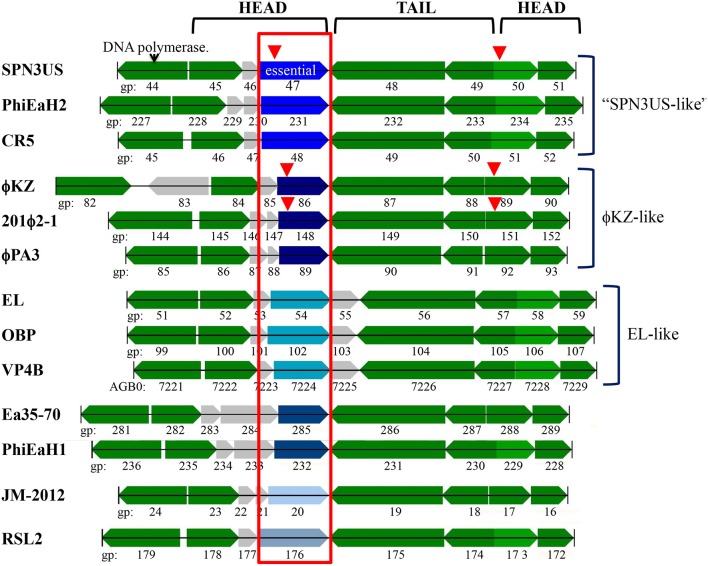
The SPN3US gene region flanking the gene encoding the essential head ejection protein gp47 and corresponding gene regions in related giant phages. Genes in the same gene location as SPN3US_0047 in related giant phages are enclosed in a red box and those shaded the same shade blue were determined to have sequence similarity by Psi-Blast. Other genes with homologs in related phages as determined by Psi-Blast searches are shaded green. SPN3US head or tail genes are indicated. Red arrowhead indicates processed by the prohead protease.

SPN3US gp47 is a low abundance head protein that, as noted above, undergoes proteolytic processing to remove an *N*-terminal propeptide of 111 residues. Interestingly, despite its essential status, gp47 did not have an easily identifiable homolog in ϕKZ. However, gp47 clearly has counterparts in the more closely related *Erwinina* phages, such as PhiEaH2 gp231, and *Cronobacter* phage CR5 gp48 (57 and 27% identity by BlastP, respectively) (Thomas et al., 2016). This led us to examine the gp47 gene locale which revealed that it is nested in a region encoding several virion protein genes which have identifiable homologs in related giant phages, all with a complex, but apparently conserved transcriptional orientations (Figure 7). Examination of this syntenous cluster indicates that the gp47 gene is in the equivalent location to that of ϕKZ gp86 and its homologs in related phages, 201ϕ2-1 gp148 and ϕPA3 gp89. Notably, the similarity between these three homologs is low, only 24–25% identity by BlastP, suggesting these genes are under selective pressures to evolve more rapidly than more highly conserved proteins, such as the terminase and major capsid proteins which have 60–69% identity by BlastP between ϕKZ, and 201ϕ2-1 and ϕPA3. Since the major capsid and terminase proteins of OBP and EL, the *Pseudomonas* phages most closely related to ϕKZ, have only 22–29% identity by BlastP to their ϕKZ homologs, we could speculate that OBP gp102 and EL gp54, whose genes are in the same locale as that of ϕKZ gp86, may have shared ancestry with gp86, but are now passed the horizon of search detection limits. Supporting this possibility is that OBP gp102 and EL gp54 have sequence homology (21% identity by BlastP).

Notably, ϕKZ gp86 is an inner head protein that undergoes processing at SQE-122 by the prohead protease gp175 and is expected to be ejected with the DNA into the host cell with its multi-subunit RNAP and other head ejection proteins (Thomas et al., 2012). Similarly, the 201ϕ2-1 homolog to ϕKZ gp86 (gp148) is processed at SQE-113 and is expected to be ejected into the host cell with its multi-subunit RNAP and other head ejection proteins (Thomas et al., 2010). In ϕKZ and 201ϕ2-1, the N-terminal fragments of gp86 and gp148 are completely cleared from the head, in contrast to the *N*-terminal fragment of gp47 of SPN3US which was detected in the lowest molecular mass gel slice. Additional experiments are needed to determine whether the *N*-terminal fragment that remains in the SPN3US head is of biological significance or if it is unable to exit the head.

Similar to the vRNAP mutants (am101), the gp47 mutant, *47*(am1), produced non-viable particles when propagated under non-permissive conditions although its virions appeared intact as judged by TEM (Figure 6B). Mass spectrometry analysis revealed that all proteins identified in the wild-type particles were present in *47*(am1), with the exception of gp172, a protein that was detected at low abundance in the wild-type phage. The lack of detection of gp172 in these mutants may be biologically relevant, or possibly it was not detected if the low molecular weight region of the gel was not included in the analyses. At 7 kDa, gp172 is the smallest SPN3US virion protein. Further experiments are required to resolve its status. Additionally, very low amounts of three proteins (gps 100, 122, and gp158) were identified in the *47*(am1) proteome that were not detected in the wild-type virion (Table 2). As the predicted function of gp100 is a thymidylate synthase, it seems unlikely that it and the other two proteins identified only in this mutant are true virion proteins. We suspect these proteins were non-specifically associated with the virion and possibly inside the capsid during head assembly. Detection of these proteins is due in part to the exceptional detection limits of the Orbitrap Fusion Lumos mass spectrometer that was used for the analyses of this mutant and *218*(am101).Erwi.

Surprisingly, we detected gp47 in the *47*(am1) particles, however inspection of its sequence coverage revealed that it ended 16 residues prior to residue Q482 whose codon is mutated to a stop codon (whereas in the wild-type virion there is coverage to K561) (Figure 4). In addition, the mass spectra identified in this mutant revealed that the proteolytic processing of the truncated form of gp47 was incomplete relative to that which we observed in the wild-type phage (Figure 4). From this we inferred that the *N*-terminal propeptide of gp47 likely has a role in targeting the protein into the prohead, similar to the shorter T4 CTS propeptides. Presumably, the incomplete processing of the gp47 propeptide and/or the absence of its *C*-terminus alters the structure and function(s) of gp47 and produces the non-viable phenotype. Based on our analyses showing that the SPN3US virion effectively assembles as a wild-type particle in *47*(am1), we infer that like the vRNAP subunits, gp47 is an ejection protein. Consistent with such a role, the mature fragment of gp47 has an *N*-terminal transmembrane domain, as predicted by TMHMM (Krogh et al., 2001) (Figure 4) which we speculate interacts with the host membrane during infection. It is interesting to note that if gp47 has a direct host-based function, possibly interacting with the host cell, this may explain the highly diverged set of genes in the equivalent position in related phages which have all evolved based on selective pressures that are a consequence of their own host interactions, akin to the gene plasticity that is seen in tail fiber proteins which have direct interactions with host cell wall components. Our analyses indicate gp47 is an excellent candidate for an inner head protein that is ejected into the host cell, possibly with a role in host takeover.

**Figure 8:**
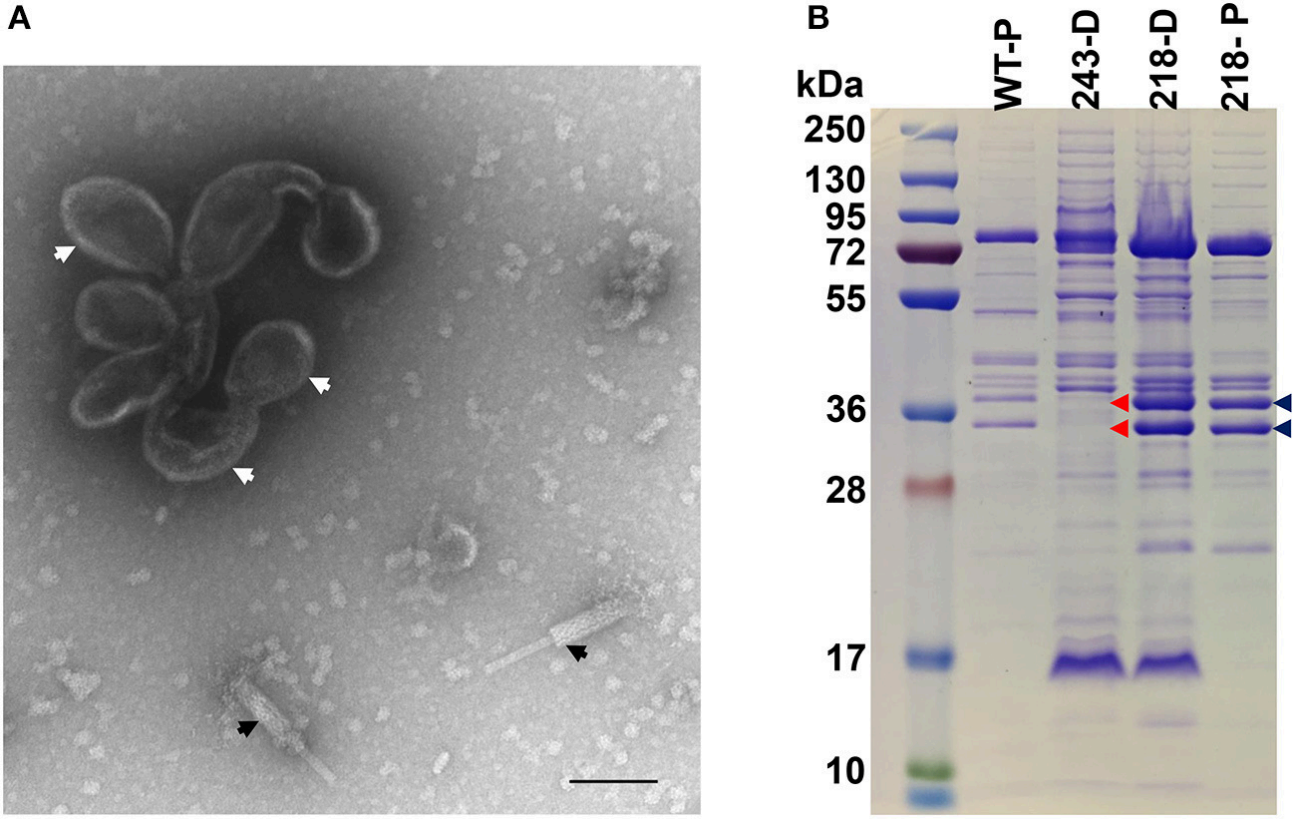
SDS-PAGE profile and morphology of SPN3US mutant *243*(am114). **(A)** Transmission electron microscopy of *243*(am114) after propagation on non-permissive *S. enterica* serovar Typhimurium (strain TT9079) and concentration by differential centrifugation. White arrows indicate disformed, DNA-empty capsid structure, black arrows indicate contracted tails, **(B)** SDS-PAGE gel showing profiles of wild-type (WT) SPN3US and amber mutant phages *243*(am214) and *218*(am101) indicated by their mutated gene names, 243 and 218, respectively, after propagation on the non-permissive strain of *S. enterica* serovar Typhimurium (TT9079). D, indicates particles concentrated by differential centrifugation; P, indicates particles purified by step and buoyant density CsCl ultracentrifugation; Blue arrows indicate the high abundance ejection proteins gp53 and gp54; Red arrows highlight the absence of the high abundance ejection proteins gp53 and gp54 in *243*(am114).

In contrast to gp47, gp243 is a medium abundance head protein that is not processed by the prohead protease and its general location in the head (i.e., shell vs. internal protein) was unknown. When *243*(am114) was propagated under non-permissive conditions, non-viable particles again were produced; however, a phenotype that is remarkably different to that of the ejection protein mutants described above was observed. The gp243- particles did not survive the first ultracentrifugation in a CsCl step gradient (i.e., no band was produced despite similar yields of sample being loaded onto the gradient), unlike the ejection protein mutants whose particles were stable through both the step and overnight buoyant density gradient ultracentrifugations. Examination by TEM of gp243-particles that had been concentrated by differential centrifugation revealed that there were no intact virions (heads joined to tails) but there were numerous free tails and apparently non-stable head structures (Figure 8A). The presence of a structure related to the head is supported by the detection of a band at the appropriate position for the MCP by SDS-PAGE in this sample (Figure 8B). Notably, in this sample there were no SDS-PAGE bands observed in the normal positions of the high abundance head proteins gps 53 and 54. From this we infer that gp243 has a function related to the incorporation of gp53 and 54 into the prohead and that without some, or all of gps 243, 53, and 54, the SPN3US head becomes highly unstable. We tentatively assign gp243 as an ejection protein, as it must interact in some manner with gps53 and 54, which are internal head proteins based on their homologs in ϕKZ being components of its large inner body (Thomas et al., 2012).

### Identification of a diverged homolog to the SPN3US prohead protease

A PSI-BLAST search of the SPN3US protease gp245 against the nr and env_nr databases identified the prohead protease homologs in other giant phages, including ϕKZ gp175, the type peptidase for MEROPs family S80 (Rawlings et al., 2012). What was unexpected was that in the second iteration of this search there was a weak match to another SPN3US protein, gp117 (1e-13 in round 4). Notably, the T4 protease gp21 was also identified in this iteration, scoring 2e-16, as were gp21 homologs in many T4-related phages including IME08 (2e-16) and *Synechococcus* phage S-PM2 (1e-34), suggesting the likelihood of non-homologous matches in the profile to be low. The identification of these matches demonstrates how sequence-to-profile based searches have gained power due to increased numbers of sequences in the databases since the ϕKZ protease had to be identified using Hidden Markov Model HMM-based strategies (Thomas et al., 2012).

A reverse PSI-BLAST search from SPN3US gp117 initially found homologs to proteins in phages that infect *E. amylovora*, such as Stratton gp135 and Kwan gp144, none of which were their assigned prohead protease (e.g., Stratton gp267 and Kwan gp271). On examination, these proteins were also identified with comparable scores to gp117 in the PSI-BLAST searches from SPN3US gp245. In the third and later iterations, matches to the biochemically validated prohead proteases of SPN3US and ϕKZ and their homologs in related phages (e.g., 201ϕ2-1 gp268, PhiPA3 gp205, *Vibrio* phage JM-2012 TSMG0080) were drawn into the profile. Further inspection revealed that several phages, in addition to SPN3US and the *Erwinia* phages, had a diverged match to their prohead proteases, such as 201ϕ2-1 gp206 (145 aa), PhiPA3 gp143 (136 aa).

The presence of a paralogous gene to its prohead protease in a phage genome was unprecedented in the literature to our knowledge, so we sought to further test the match between SPN3US gp117 and known prohead proteases using HMM-based strategies. First, an alignment was made between gp117 and 12 homologs from 9 *Erwinia* phages with the Sequence and Alignment Modeling software (SAM) (Hughey and Krogh, 1996; Hughey et al., 2003). (Note that there are two homologs to gp117 in the phages Machina, Caitlin and ChrisDB.) An HMM based on this alignment was scored against a library of all SPN3US proteins, and the prohead protease gp245 had an E-value of 2.4e-09. Conversely, an HMM based on the SPN3US protease gp245 aligned with its homologous proteases in 24 related phages scored gp117 at 1.50e-09. Although the E-values in our searches are inflated as the result from searches of small libraries, the identification of a false positive seems less likely when two different models are each able to find the protein of interest in reverse searches.

To further interrogate the validity of SPN3US gp117 having similarity to gp245 and other known proteases, we used HHpred for its sensitive profile-to-profile (HMM-HMM) based searches (Söding, 2005; Söding et al., 2005). An HHsearch of the gp245 HHM against the T4 gp21 HHM used originally to identify the ϕKZ protease gp175 (Thomas et al., 2012) gave an E-value of 1.1e-11 and an alignment with the three residues of the catalytic triad of T4 gp21 was found (gp245 residues H-77, S-153, D-178). An HHsearch of the gp117 HHM against this T4 HHM gave an E-value 4e-07 and aligned two of the three catalytic residues in the T4 enzyme to gp117 residues S-126 and D-144. Notably, an HHsearch of the two SPN3US HHMs against one another gave an E-value of 3e-17 and also aligned the catalytic serine and aspartate of gp245 with gp117 residues S-126 and D-144. While there was no direct alignment between gp117 and the catalytic histidine of the two known protease HHMs, there is a histidine seven residues upstream in gp117.

## Discussion

### The SPN3US head structure is architecturally T4 gone rococo

**Figure 9:**
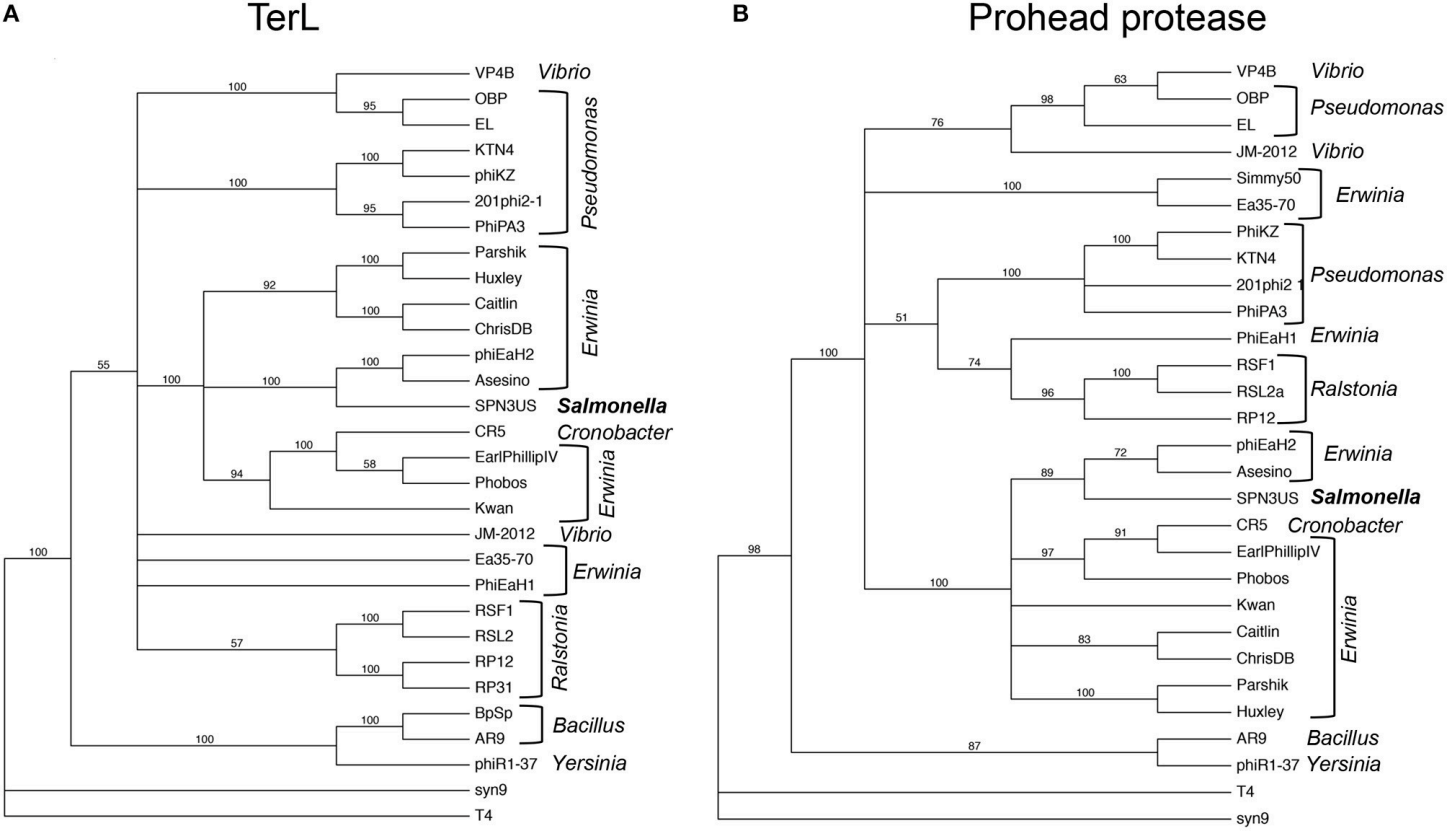
Phylogenetic trees of phage head maturation enzymes in SPN3US and related giant phages. **(A)** Phylogentic tree of the large terminase subunit (TerL), and **(B)** phylogenetic tree of the prohead protease. T4 and the T4-related phage Syn9 proteins were used to root the trees. Phage hosts are indicated.

Our analyses of the SPN3US head highlight major themes of head structure and assembly that are conserved between SPN3US, related giant phages, and T4. Critically, the proteins identified in T4 as essential for the formation of its large myoviral capsid shell (major capsid protein and portal) clearly have homologs in SPN3US despite high divergence at the sequence level. SPN3US and related phages all also have homologs to the two essential enzymes in T4 required for head maturation and DNA packaging—the prohead protease and the large terminase protein, respectively (Figure 9). Despite the absence of identifiable homologs in T4 for the other SPN3US head protein, the SPN3US head does have numerous shared features with the T4 head, notably internal head proteins that are ejected into the host cell as well as paralogous proteins. The existence of paralogs in both T4 and SPN3US is intriguing as most dsDNA phage genomes do not have paralogs (Kristensen et al., 2011). That both phage genomes do contain paralogous genes is likely the consequence of a shared ancestral replication/recombination pathway as evidenced by diverged homologs in SPN3US to the T4 DNA polymerase (SPN3US gps 18 and 44 which represent a split subunit DNA polymerase as initially identified in phage OBP, Cornelissen et al., 2012) and UvsX (SPN3US gp216).

The detection of proteolysis in eight SPN3US head proteins revealed shared characteristics with the processing of head proteins that occurs in T4. Most notably, in both phages cleavage always occurs after a glutamate residue in a short motif. Our studies also revealed that processing can occur on the *N*-termini and/ or *C*-termini of head proteins in both SPN3US and T4. That both SPN3US and ϕKZ proteases undergo *C*-terminal autoprocessing highlights a need to resolve the relevance of this event in prohead assembly and maturation and/or enzyme activation. Additional biochemical studies of the T4 protease are also needed to elucidate the autocleavage mechanism of gp21 and the role of gp21 in head maturation.

A number of processed SPN3US head proteins showed evidence of multiple processing sites in their propeptide regions. Multiple processing with a substrate protein by the prohead protease is a well-known feature of the T4 MCP propeptide as well as core proteins, such as gp22. We also observed multiple processing sites in ϕKZ head proteins (Thomas et al., 2012; Thomas and Black, 2013). Presumably, this aids in ensuring that these peptides are cleared from the capsid either before or during head expansion/DNA packaging. To comprehensively define the heterogeneity of processing sites for each protein species is beyond current proteomic capabilities. Other analyses are needed to more accurately determine the number and location of the cleavage sites, such as conducted for ϕKZ gp93 (Thomas and Black, 2013). Importantly, the identification of similarities in processing between SPN3US and T4 and also SPN3US and ϕKZ and 201ϕ2-1 provide support for our conclusion that processing by a prohead protease is a conserved, possibly ancient, essential step in head maturation in all related giant phages.

Just as significantly, our analyses of SPN3US have highlighted several major differences that have evolved since T4 and giant phages shared an ancient ancestor. Superficially, these include major variations in head size and structure (T = 27 for ϕKZ and SPN3US, vs. T_ends_ = 13 for the caps and T_mid_ = 20 the prolate capsid of T4, Fokine et al., 2004) in addition to numerous different head proteins in the giant phages (~50 proteins vs. 13 head proteins in T4, Black et al., 1994). The higher number of head proteins in the giant phages could be attributed to a highly complex, possibly more independent life-cycle of the giant phages, as evidenced by their vRNAPs (Ceyssens et al., 2014) and the large number of head ejection proteins, some of which, such as gp47, likely have roles in host takeover. However, it is also feasible that the high number of head proteins in SPN3US may be, to some extent, a consequence of a genome framework that allows rampant gene duplication and recombination events and that many of these proteins are not essential. We anticipate that this question will be resolved through further analysis of our SPN3US mutant collection to identify all essential head genes.

Additionally, our in-depth analyses of proteolytic processing during head maturation has revealed distinct variations between SPN3US and its relatives, vs. T4. The giant phages have portal proteins with a massive propeptide, unprecedented not only in T4 but any other phage taxon. In addition, the giant phages all have multiple inner head proteins that have propeptides that are much longer than the 10–20 residue propeptide CTS of the T4 internal proteins and Alt. In T4, the CTS functions to ensure that each protein is incorporated into the prohead; the propeptide is then removed from the protein via proteolysis and presumably escapes from the head through small pores in the shell during maturation (Mullaney and Black, 1996). The T4 CTS is so effective at its targeting role that it has been used to package numerous proteins of non-phage origin into T4 heads (Mullaney and Black, 1998; Mullaney et al., 2000). Based on our analyses of SPN3US, particularly of the gp47- mutant into which a *C*-terminally truncated protein was packaged into the head, it is likely the giant phage propeptides have similar functions to that of the T4 CTS sequences. The need for longer propeptides and whether any of the SPN3US propeptides have an additional role, such as core/scaffold formation is yet to be determined although the latter is an important consideration for future studies since a counterpart to the T4 scaffold protein gp22 has not been confirmed in any giant phage.

### Giant phage head structure/function follows a virtuosic evolutionary pathway

A major goal of our study of SPN3US was to characterize its virion comprehensively as a foundation for further studies that implement it as a genetic model for understanding giant phage biology. As such, this new system has revealed new information regarding the functions and assembly mechanisms of core head proteins, such as the vRNAP for which homologs exist in every related giant phage (Skurnik et al., 2012; Ceyssens et al., 2014; Yakunina et al., 2015). For instance, the SPN3US system has confirmed the essential nature of the vRNAP based on our isolation of mutants in genes encoding three of the subunits, including the recently identified “missing” *C*-terminus of the β′ subunit, and in doing so supported an unprecedented scenario in tailed phage head assembly that this enzyme complex assembles as a multimer prior to incorporation into the prohead. This is truly remarkable when one considers that the packaged vRNAP must then undergo subsequent major conformational rearrangements in the DNA packed capsid to allow for its ejection through the ~30 Å diameter tail tube into the *Salmonella* cell, where it must then reassemble to be able to transcribe the injected phage DNA. Our vRNAP finding raises the question as to whether the vRNAP multimer is active prior to incorporation into the prohead and highlights a need for further study on this remarkable complex. In addition, our new genetic system has facilitated the characterization of essential head proteins gps 47 and 243, including that the function of gp47 is related to host infection/takeover and that gp243 has a role in the incorporation of members of the paralog family A, for which there are counterparts in all related phages.

Our analyses of SPN3US have revealed unexpected, almost virtuosic, aspects of giant phage head composition and assembly. Regarding composition, the most obvious examples are the head paralog families which show remarkable plasticity in numbers between different phages but also variations in abundance within the same phage. For instance, the numbers of proteins belonging to paralog family A containing the PFAM domain 12699 has been shown to vary between two members (e.g., SPN3US, this study) to seven members (e.g., phiPA3, Cornelissen et al., 2012), while the numbers of paralog family B proteins varies between two (e.g., ϕKZ, Mesyanzhinov et al., 2002) to 20 members in SPN3US. Our estimate of the copy numbers of the processed paralog family A members (>600 copies each per virion) was much higher than our estimates for any of the ϕKZ inner head proteins (Thomas et al., 2012). This was unexpected, as it implies the combined molecular mass of these two 31-kDa proteins in the SPN3US head (>40 MDa) is at least double the estimate of the molecular mass of the ϕKZ IB (Thomas et al., 2012; Wu et al., 2012) and highlights a need for further research to more rigorously quantify internal head protein copy numbers, not only in SPN3US but other related giant phages.

The need for further studies to more accurately quantify the copy numbers of ejection proteins in giant phage heads is additionally underscored by the fact that the SPN3US genome is ~40 kb shorter than that of ϕKZ. Both SPN3US and ϕKZ likely have the same headful packaging strategy as T4 as their DNAs are packaged to about the same density within their capsids and their large terminase proteins have homology to that of T4, gp17 (Figure 9). In T4, DNA packaging concludes when gp17 cleaves the concatemeric DNA by sensing that the capsid is completely full of DNA (Black, 2015). That is, cleavage by the packaging motor is not based on genome length or sequence specificity, but rather reflects the DNA density within the head. Hence, if there were additional, or conversely, less ejection proteins in a phage's capsid, its terminase would accommodate by packaging less or more genomic DNA, respectively. Further research is required to clarify the roles of different ejection proteins in giant phages and also to test our hypothesis that there is a relationship between ejection protein abundance, DNA packaging and genome length in giant phages.

Our studies also highlighted an unexpected plasticity in the proteolysis maturation step among different giant phages. Despite the fact that the SPN3US head is composed of similar numbers of proteins as 201ϕ2-1 and ϕKZ, we found that proteolytic processing occurs during head maturation in only eight SPN3US head proteins, in contrast to the 19 processed proteins in both 201ϕ2-1 and ϕKZ (Thomas et al., 2010, 2012). Initially, we attributed this difference to variations in protein composition between the phages, but based on our correlation of MS sequence coverage and gel migration of individual SPN3US proteins in the wild-type and numbers of mutant proteomes these explanations do not fully account for the reduced number of processed proteins in SPN3US vs. 201ϕ2-1 and ϕKZ.

**Figure 10:**
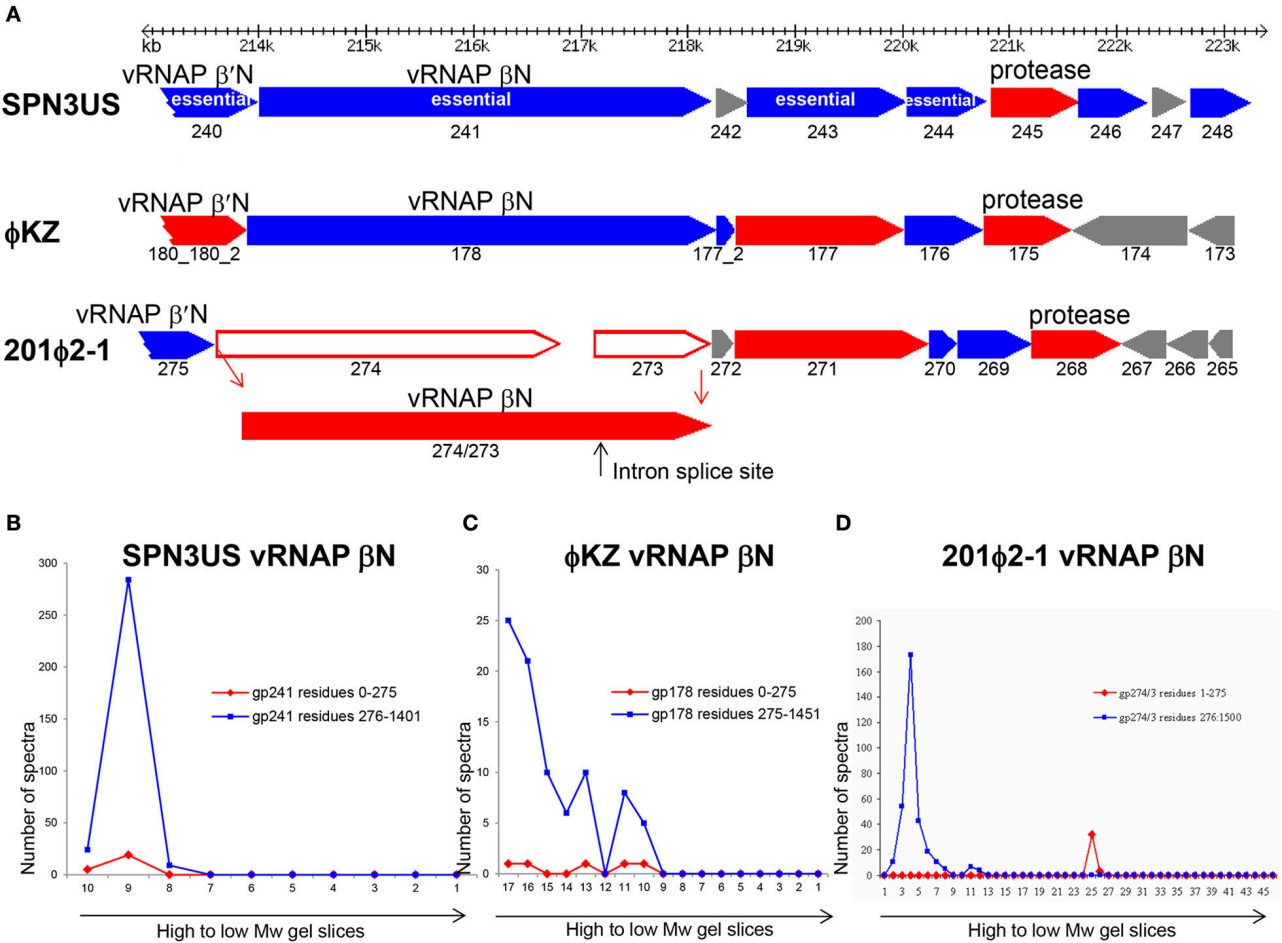
Comparison of proteolytic processing in homologous SPN3US, ϕKZ and 201ϕ2-1 proteins **(A)** Gene region encoding four essential head proteins including the vRNAP βN subunit in SPN3US and corresponding regions in ϕKZ and 201ϕ2-1. Red arrows indicate genes encoding virion proteins processed by the prohead protease, blue arrows indicate genes encoding virion proteins not processed by the prohead protease. Gray arrows indicate non-virion genes. Plot of total mass spectra detected in SDS-PAGE gel slices by mass spectrometry for the RNAP βN subunit of **(B)** SPN3US, **(C)** ϕKZ, and **(D)** 201ϕ2-1.

The variability in the proteolytic processing status of homologous proteins in SPN3US, 201ϕ2-1, and ϕKZ is illustrated in the products of a syntenous head gene region which includes the vRNAP β subunit and protease genes (Figure 10). Although the prohead protease in all three phages undergoes auto-proteolysis, the processing status of other proteins from this region is variable. For instance, the 201ϕ2-1 vRNAP β subunit is processed by removal of an *N*-terminal propeptide (cleaved at TFE-275) (Thomas et al., 2010) of similar length to the long propeptide removed from the portal proteins of all three phages (Figure 10). Strikingly, our evidence clearly indicates that the SPN3US vRNAP β subunit is not processed (Supplementary Figure 2). We also believe that the ϕKZ homolog gp178 is not processed, but since there were about 10-fold less spectra identified for gp178 than either its 201ϕ2-1 or SPN3US counterparts, this conclusion requires confirmation.

We found no evidence to support proteolytic processing of several other SPN3US proteins although their homologs in other giant phages are processed. For instance, SPN3US essential protein gp243 is not processed (204 total spectra were detected giving an overall coverage of 78%) (Supplementary Figure 3) but 201ϕ2-1 gp271 and ϕKZ gp177 are both cleaved by their prohead proteases at AVE-61 and SVE-60, respectively. Similarly, essential head protein gp214 of SPN3US (Thomas et al., 2016) is not processed (Supplementary Figure 3) whereas both its ϕKZ homolog, gp153, and 201ϕ2-1 homolog, gp238, are processed at SQE-52 and STE-64, respectively.

The variability in the proteolytic processing of head proteins we observed in different giant phages was unexpected because we had assumed that all the internal head proteins of the giant phages would undergo proteolytic processing, because they share essential assembly steps with T4 and all T4 internal head proteins are processed. Also, we had expected that any essential head protein with a conserved function in a giant phage would likely go through the same assembly and maturation processes in related giant phages. That neither expectation held true leads to the conclusion that for numerous giant phage head proteins, there are no negative consequences in terms of protein function/phage viability if the regions considered as propeptides in their counterparts in related phages are not removed by proteolysis. There is probably no better illustration of this than the SPN3US vRNAP β subunit which functions with the long *N*-terminal region still attached, although it is feasible that the retention of this domain affects enzymatic activity/specificity relative to the RNAPs of other giant phages in which it is removed, such as 201ϕ2-1 gp274/3.

A major question arising from our observations of major variations in processing of head proteins in giant phages is “What were the forces that led to these variations?” Did mutations in the protease gene alter enzyme specificity and, therefore, influence which proteins could be processed? Indirect evidence that this may have occurred is that the SPN3US protease has a narrower cleavage sequence specificity (A-X-E) relative to that of its counterparts in 201ϕ2-1 (S/A/G/T-X-E, with 2 A-X-E processing sites) and ϕKZ (S/A/G-X-E, with eight A-X-E processing sites). Additionally, did an event(s) affecting the protease gene alter protease function? Support that such an event, may have occurred can be found in our identification of an extremely diverged match to the SPN3US and T4 proteases, gp117. Notably, the equivalent to the 3′ end of the gp245 protease gene is absent in the gp117 gene, although the downstream gene gp118 is an appropriate length if fused with gp117 to form a protein of almost identical length to that of gp245. In gp245 we infer it is its *C*-terminal region that targets the enzyme into the prohead because it is removed via auto-proteolysis (Figure 4). Genetic analyses of T4 showed that if the protease is not incorporated into the prohead, head morphogenesis is effectively frozen and no viable virions are produced (Showe et al., 1976a,b). If we assume that the removal of the *C*-terminal region of a giant phage protease would have a similarly disastrous outcome, then, logically, that phage could only form viable progeny after a protease gene truncation event in one of two ways: 1. if proteolytic processing of proteins was not essential and/or 2. if the phage acquired a version of a protease gene that encoded an active enzyme with similar packaging and substrate specificities as the original enzyme. The latter could occur via a duplication event within the same genome or a recombination event with a related phage. While we can only speculate about the existence of both a protease gene and a potential protease remnant in the SPN3US genome, there is excessive evidence within its genome, and those of related giant phages, that gene splitting, duplication and recombination events have abounded during their evolution making such scenarios as described not implausible. In addition, Liu and Mushegian (2004) demonstrated that displacement of protease genes has occurred many times within the order *Caudovirales*, albeit on a broader scale between proteases with different enzymatic specificities, Herpesvirus-like proteases and Clp-like proteases (Liu and Mushegian, 2004).

We conclude that the prohead proteases in both T4-like and giant phages have remarkable functions, cleaving thousands of head proteins in just a few minutes to facilitate a major remodeling of the prohead prior to DNA packaging. Our study highlights that there is still much to be learned about the prohead proteases in both giant phages and T4. However, the variations we have observed in head protein proteolysis between different phages indicate that head maturation has undergone myriad evolutionary events. Consequently, giant phage proteases have likely had a greater impact on giant phage head assembly, structure, composition and possibly even genome length than previously realized.

## Author contributions

JT conceived and supervised the project. JT, BA, MD, SM, AB, and LWB performed experiments and data analyses. SW supervised the MS analysis and performed the MS data analysis. LJB and MO assisted with bioinformatics analyses. All authors read and approved the final manuscript.

### Conflict of interest statement

The authors declare that the research was conducted in the absence of any commercial or financial relationships that could be construed as a potential conflict of interest.
