# Supplementary material for: To Be or Not To Be T4: Evidence of a Complex Evolutionary Pathway of Head Structure and Assembly in Giant Salmonella Virus SPN3US
Source: Front Microbiol. 2017 Nov 15;8:2251. doi: 10.3389/fmicb.2017.02251 (PMC5694885; doi:10.3389/fmicb.2017.02251)
Supplement: Supplementary file 1 [file Table1.PDF]

Supplementary Table 1. Virion proteins in purified wild-type SPN3US identified by mass spectrometry with a protein identification probability of 100 %. Proteins are ordered from high to low relative abundance as determined from spectral counts adjusted for molecular weight (SC/M). Proteins identified as part of the head are noted.

gp	Slice[1]	Mass, kDa (Proc.[2])	Unique Peptides	Total SC[3] (MudPit)	SC/M[4] (processed)	Percentage sequence coverage	Paralog family	Comment (expected copies per virion)
75	7	83.9 (70.4)	144	1592	18.97(22.61)	76		**Major capsid** (1560 copies) Processed ATE-130
53	3	45.2 (31.5)	58	662	14.65 (21.02)	65	A	Head - IB protein candidate. Processed AQE-125
54	3	45.1 (31.9)	56	643	14.26 (20.16)	57	A	Head- IB protein candidate. Predict Processed AQE-124
160	1	18.5	38	175	9.46	89	B	Head
141	4	32.6	54	306	9.39	94	B	Head
256	7	75.7	71	669	8.84	85		**Tail sheath** (expect ~264 copies)
255	4	32.7	21	209	6.39	73		**Tail tube** (expect ~264 copies)
243	6	54.6	55	251	4.60	77		Head
143	3	31.9	17	118	3.70	92	B	Head
74	1	12.9	11	40	3.10	59		Head
45	5	50.3 (48.2)	37	144	2.86 (2.99)	57		Head - Processed ASE-20
257	4	34.2	14	97	2.84	57		Head
214	2	28.1	19	79	2.81	70		Head
144	2	30	25	83	2.77	75	B	Head
237	1	19.9	8	50	2.51	64	B	Head
51	3	34.9	20	81	2.32	53		Head
109	1	17.7	12	40	2.26	93		Head
149	4	36.2	19	78	2.15	83	B	Head
142	3	30.8	18	65	2.11	80	B	Head
167	5	44.8	25	85	1.90	70		

259	6	61	29	115	1.89	69		Head
152	4	36.8	23	69	1.88	86	B	Head
47[5]	5	62.8 (50.7)	31	110	1.75 (2.17)	55		Head Processed AVE-79
84	4	32.9	14	51	1.55	52		
83	1	20	8	30	1.50	40	B	Head
52	1	21	11	30	1.43	55		Head
50	2	39.4 (25.6)	21	56	1.42 (2.19)	43		Head Processed ATE-127
169	9	149	66	210	1.41	62	C	
203	5	51.9	23	72	1.39	53		
202	2	23.5	10	32	1.36	60		
140	3	31.4	15	41	1.31	65	B	Head
139	3	29.8	10	37	1.24	54	B	Head
138	3	29.2	13	35	1.20	78	B	Head
241	9	159.1	71	189	1.19	61		Head **vRNAP β′N**
48	9	111	45	125	1.13	67		
168	10	188.1	65	203	1.08	49	C	
218	2	25.2	13	27	1.07	39		Head **vRNAP βC**
8	2	30.3	10	32	1.06	55		Head
245	2	30.7 (23.4)	9	25	0.81 (1.06)	30		Head **Prohead protease**, Processed at AQE-203
42	5	49.3	19	52	1.05	66		Head **vRNAP β′M**
46	1	16.2	6	17	1.05	28		Head
170	9	135.4	51	135	1.00	66	C	
148	6	53.7	25	53	0.99	65	B	Head
81	7	100.2 (72.3)	27	93	0.93 (1.29)	47		Head **Portal (12 copies)** Processed ATE-161, expected maturation is AQE-254
246	2	23.9	13	22	0.92	61		Head
145	6	50.8	16	46	0.91	50	B	Head
154	5	50	19	45	0.90	68	B	Head
262	5	52.7	23	46	0.87	54		Head

150	3	33.8	9	29	0.86	36	B	Head
172	1	7	4	6	0.86	81		
95	1	17.6	8	15	0.85	56		
17	1	15.3	6	13	0.85	48		
62	6	52	18	44	0.85	56		
94	4	41.6	19	35	0.84	48		Head, could be proc AME-60
146	4	36.9	10	31	0.84	45	B	Head
248	1	21	10	17	0.81	68		Head
258	8	96.4	36	76	0.79	54		
239	10	259.1	107	200	0.77	37		
153	3	33.8	7	26	0.77	50	B	Head
193	1	19.9	9	15	0.75	54		Head
171	5	47.6	17	35	0.74	46		
155	8	78.3	28	55	0.70	60		Head
242	1	10.5	5	7	0.67	50		Head
124	9	113.7	39	73	0.64	48		
223	5	45.3	17	29	0.64	43		
61	6	58.3	20	35	0.60	52		
97	1	11.9	4	7	0.59	32		Head
238	8	82.1	24	46	0.56	43		
225	2	25.1	11	14	0.56	41		Head
240	6	59.6	23	33	0.55	35		Head **vRNAP β′N**
91	2	23.6	7	13	0.55	51		Head
147	3	33.7	6	17	0.50	42	B	Head
21	4	40.7	11	20	0.49	34		Head
98	2	22.4	7	10	0.45	43		
244	2	27	7	12	0.44	46		Head **vRNAP β′C**
151	6	52.2	13	25	0.48	34	B	Head
82	8	84.4	22	36	0.43	42		
123	1	16.5	5	7	0.42	32		
25	1	14.6	4	6	0.41	42		

64	5	48.9	13	20	0.41	48		
157	2	24.5	4	9	0.37	25		Head
37[6]	1	14.6	4	5	0.34	43		
41	3	31.8	4	8	0.25	26		
173	4	34.6	6	8	0.23	23		
38	2	23.2	3	4	0.17	26		
49	5	48.1	5	7	0.15	17		

1 – The slice in the SDS-PAGE gel (Fig. 1) in which the spectral count for each protein peaked.
2 – "Proc." indicates molecular weight after processing by the prohead protease, gp245.
3 – "SC" represents the total number of spectral counts for each protein, as determined by the mass spectrometric MudPIT analyses
4 – "SC/M" indicates total spectral count summed over all slices adjusted by molecular mass. Numbers provided in parentheses are the total spectral count adjusted by the processed molecular mass.
5 – Mass spectral analyses re-assigned the start site of the gp47 gene to at nucleotide position 44887 in JN641803.1 which has additional four codons to the predicted start site. Processing sites of the prohead protease in gp47 are for the new peptide co-ordinates (see text).
6 – gp37 was not identified in the MudPIT analysis at 3 minimum peptides, but was identified in the single slice data; with peptide identification probability of 100 % for 4 of 5 spectra and of 99 % for an additional spectra.
