# Supplementary material for: To Be or Not To Be T4: Evidence of a Complex Evolutionary Pathway of Head Structure and Assembly in Giant Salmonella Virus SPN3US
Source: Front Microbiol. 2017 Nov 15;8:2251. doi: 10.3389/fmicb.2017.02251 (PMC5694885; doi:10.3389/fmicb.2017.02251)
Supplement: Supplementary file 2 [file Table2.PDF]

Supplementary Table 2. Mutations identified in SPN3US amber mutant phages.

Mutant	Reference Position	Type	Reference Base	Called Base	Impact	SNP %	P not ref	Q call	Feature Type	Feature Name	DNA Change	Amino Acid Change
**am84**	77962	Ins	-	T		94.80%	100.00%	60			g.77962insT	
	80448	SNP	G	A	Synonymous	99.80%	100.00%	60	CDS	SPN3US_0081	c.268C>T	p.(=)
	87715	Ins	-	C		88.50%	100.00%	60			g.87715insC	
	93451	Ins	-	T		90.80%	100.00%	60			g.93451insT	
	193250	SNP	G	A	Synonymous	100.00%	100.00%	60	CDS	SPN3US_0223	c.177G>A	p.(=)
	200944	Ins	-	T		97.10%	100.00%	60			g.200944insT	
	220491	SNP	C	T	Nonsense	99.80%	100.00%	60	CDS	SPN3US_0244	c.469C>T	p.Q157.
	223871	SNP	C	A	Non-synonymous	99.80%	100.00%	60	CDS	SPN3US_0250	c.70C>A	p.R24S
	239933	Ins	-	T		95.70%	100.00%	60			g.239933insT	
**Mutant**	**Reference Position**	**Type**	**Reference Base**	**Called Base**	**Impact**	**SNP %**	**P not ref**	**Q call**	**Feature Type**	**Feature Name**	**DNA Change**	**Amino Acid Change**
**am101**	37508	SNP	C	T	Synonymous	99.80%	100.00%	60	CDS	SPN3US_0039	c.1455C>T	p.(=)
	58847	SNP	G	A	Non-synonymous	99.80%	100.00%	60	CDS	SPN3US_0059	c.127G>A	p.A43T
	63479	SNP	G	A	Synonymous	99.80%	100.00%	60	CDS	SPN3US_0064	c.393G>A	p.(=)
	64780	SNP	G	A	Non-synonymous	100.00%	100.00%	60	CDS	SPN3US_0065	c.337G>A	p.A113T
	77962	Ins	-	T		93.70%	100.00%	60			g.77962insT	
	87715	Ins	-	C		84.80%	100.00%	60			g.87715insC	
	93451	Ins	-	T		90.60%	100.00%	60			g.93451insT	
	146012	SNP	C	T	Non-synonymous	100.00%	100.00%	60	CDS	SPN3US_0168	c.4708G>A	p.A1570T
	189811	SNP	C	T	Nonsense	99.80%	100.00%	60	CDS	SPN3US_0218	c.49C>T	p.Q17.
	196211	SNP	C	T	Non-	100.00%	100.00%	60	CDS	SPN3US_0226	c.298C>T	p.P100S

					synonymous							
	200944	Ins	-	T		97.00%	100.00%	60			g.200944insT	
	223871	SNP	C	A	Non-synonymous	99.70%	100.00%	60	CDS	SPN3US_0250	c.70C>A	p.R24S
	231277	SNP	C	T	Synonymous	99.80%	100.00%	60	CDS	SPN3US_0258	c.1119C>T	p.(=)
	239933	Ins	-	T		97.60%	100.00%	60			g.239933insT	
**Mutant**	**Reference Position**	**Type**	**Reference Base**	**Called Base**	**Impact**	**SNP %**	**P not ref**	**Q call**	**Feature Type**	**Feature Name**	**DNA Change**	**Amino Acid Change**
**am114**	111268	SNP	C	T	Synonymous	99.80%	100.00%	60	CDS	SPN3US_0128	c.81C>T	p.N27N
	151685	SNP	C	T	Synonymous	99.80%	100.00%	60	CDS	SPN3US_0169	c.3261G>A	p.A1087A
	154174	SNP	C	T	Non Synonymous	99.70%	100.00%	60	CDS	SPN3US_0169	c.772G>A	p.V258I
	180070	SNP	G	A	Synonymous	100.00%	100.00%	60	CDS	SPN3US_0204	c.54G>A	p.G18G
	213107	SNP	C	T	Synonymous	99.80%	100.00%	60	CDS	SPN3US_0240	c.751C>T	p.L251L
	218592	SNP	C	T	Nonsense	99.50%	100.00%	60	CDS	SPN3US_0243	c.61C>T	p.Q21.
	223871	SNP	C	A	Non Synonymous	100.00%	100.00%	60	CDS	SPN3US_0250	c.70C>A	p.R24S
