# Supplementary material for: To Be or Not To Be T4: Evidence of a Complex Evolutionary Pathway of Head Structure and Assembly in Giant Salmonella Virus SPN3US
Source: Front Microbiol. 2017 Nov 15;8:2251. doi: 10.3389/fmicb.2017.02251 (PMC5694885; doi:10.3389/fmicb.2017.02251)
Supplement: Supplementary file 3 [file Table3.PDF]

Table 3 Mass spectrometry of SPN3US amber mutant phages after purification via ultracentrifugation in CsCl step and CsCl equilibrium gradients. The total spectral count for each SPN3US protein was calculated from MudPIT analyses. Note that am101 and am1 underwent analyses on a Thermo Fisher Orbitrap Fusion Lumos/ETD mass spectrometer, whereas am84 underwent analyses on an Orbitrap mass spectrometer.

gp	Mw, kDa	*244*(am84)			*218*(am101)			*47*(am1)		
		Unique peptides	Total spectral count	Percent sequence coverage, %	Unique peptides	Total spectral count	Percent sequence coverage, %	Unique peptides	Total spectral count	Percent sequence coverage, %
8	30.3	10	19	55	14	59	77	12	45	74
17	15.3	9	17	71	7	16	56	6	12	51
21	40.7	14	21	41	16	32	51	15	38	51
25	14.6	5	8	48	5	8	42	4	12	41
28	32	0	0	0	5	7	27	5	7	27
33	12.3	3	4	35	0	0	0	3	4	41
37	14.6	0	0	0	0	0	0	4	5	43
41	31.8	0	0	0	0	0	0	8	27	47
42	49.3	0	0	0	0	0	0	23	61	67
45	48.2	27	128	59	25	94	50	32	156	59
46	16.2	9	25	55	9	38	47	10	37	49
47	50.8	27	129	52	38	164	70	33	135	59
48	111	40	117	53	47	176	53	46	239	56
49	48.1	5	5	19	10	21	42	10	21	47
50	25.6	22	88	37	28	112	39	26	112	48
51	34.9	21	104	64	29	139	58	28	154	58
52	21	13	28	44	17	48	61	18	51	67
53	31.5	63	868	61	91	1375	69	97	1507	72
54	31.9	53	776	53	87	1005	62	80	1072	61
61	58.3	10	17	29	19	58	52	21	56	60
62	52	16	38	49	24	43	61	22	50	61
64	48.9	7	8	19	17	24	60	14	32	48
74	12.9	15	47	62	19	59	70	16	56	67

75	70.4	142	1754	73	230	2664	75	265	4168	75
81	72.3	32	72	50	40	146	60	36	137	54
82	84.4	21	39	39	26	80	50	28	78	61
83	20	8	21	40	8	24	38	9	30	49
84	32.9	15	91	53	24	133	56	29	137	73
91	23.6	7	10	37	9	12	45	11	17	55
94	41.6	7	10	17	7	11	19	15	31	41
95	17.6	7	9	45	8	15	51	10	17	66
97	11.9	7	8	54	6	9	47	7	10	60
98	22.4	0	0	0	0	0	0	5	5	31
100	23.2	0	0	0	4	4	25	4	5	22
109	17.7	12	32	93	13	34	93	10	30	93
122	18.8	0	0	0	0	0	0	3	5	11
123	16.5	6	8	33	3	6	27	4	6	29
124	113.7	29	48	37	29	86	40	34	130	46
138	29.2	14	50	83	18	106	86	15	105	86
139	29.8	10	50	55	26	109	83	17	82	72
140	31.4	14	62	58	19	129	65	14	62	62
141	32.6	57	491	96	100	777	88	83	731	96
142	30.8	23	122	82	21	151	81	22	125	81
143	31.9	19	214	81	28	319	92	25	271	92
144	30	21	101	67	37	167	74	34	122	74
145	50.8	15	28	47	19	49	58	20	48	58
146	36.9	10	32	58	13	57	45	10	60	45
147	33.7	10	27	57	20	62	64	12	42	60
148	53.7	24	53	66	29	80	63	29	98	68
149	36.2	20	96	87	23	168	87	20	136	68
150	33.8	10	42	36	15	78	43	14	44	36
151	52.2	12	32	41	17	45	54	17	58	61
152	36.8	24	102	86	22	133	90	24	128	89
153	33.8	10	40	51	11	51	65	9	51	56

154	50	19	36	66	25	72	71	26	81	79
155	78.3	26	68	54	41	121	77	36	123	73
158	19.9	0	0	0	4	4	30	5	7	37
160	18.5	29	184	88	55	264	89	31	225	89
167	44.8	26	66	69	36	127	89	34	165	85
168	188.1	54	152	39	75	212	46	75	365	53
169	149	66	190	56	64	200	50	67	347	64
170	135.4	41	130	52	50	138	48	49	265	55
171	47.6	16	33	51	16	42	43	17	53	48
173	34.6	6	8	32	12	20	53	10	22	40
193	19.9	7	8	51	16	13	54	6	10	35
202	23.5	11	33	65	19	49	90	14	44	68
203	51.9	19	66	52	20	96	55	22	124	57
214	28.1	14	65	67	4	145	67	21	142	77
218	25.2	0	0	0	0	0	0	13	30	39
223	45.3	19	25	54	21	31	60	20	32	63
225	25.1	11	23	56	14	31	61	10	30	39
237	19.9	9	29	67	10	25	53	9	24	55
238	82.1	20	38	28	29	59	51	26	70	45
239	259.1	68	94	34	57	86	30	89	202	46
240	59.6	0	0	0	0	0	0	32	92	62
241	159.1	0	0	0	0	0	0	66	247	59
242	10.5	3	8	36	4	18	46	8	18	63
243	54.6	38	300	70	58	361	72	52	429	74
244	27	0	0	0	0	0	0	10	25	46
245	23.4	9	32	39	13	44	48	11	41	27
246	23.9	18	52	76	22	66	72	19	67	74
248	21	8	12	62	10	17	40	11	19	51
255	32.7	18	239	69	31	369	72	27	400	70
256	75.7	71	664	88	144	1201	90	154	1443	92
257	34.2	14	81	57	22	170	61	22	160	57

258	96.4	41	108	58	41	143	57	37	201	51
259	61	27	126	73	33	219	69	32	238	77
262	52.7	12	16	36	19	28	42	29	76	63
