# Supplementary material for: To Be or Not To Be T4: Evidence of a Complex Evolutionary Pathway of Head Structure and Assembly in Giant Salmonella Virus SPN3US
Source: Front Microbiol. 2017 Nov 15;8:2251. doi: 10.3389/fmicb.2017.02251 (PMC5694885; doi:10.3389/fmicb.2017.02251)

Supplementary Figure 1. Peptide coverage of T4 ejection proteins detected by mass spectrometry **(A)** Internal protein (IPII), **(B**) IPIII, and **(C)** Alt.   Red arrow indicates a T4 prohead protease gp21 processing site identified via a semi-tryptic peptide. Note the two processing sites in Alt had not been previously confirmed biochemically.   Green arrow indicates a T4 prohead protease gp21 processing site determined previously.

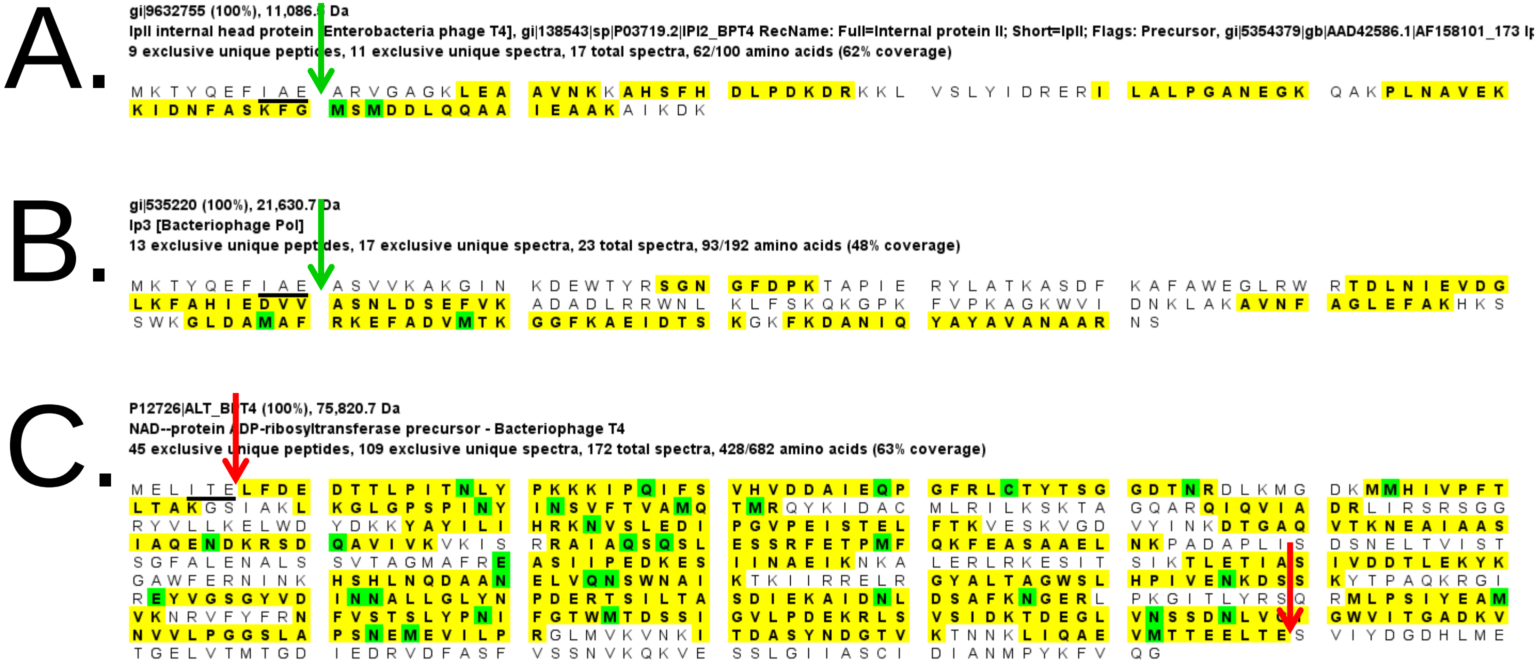

Supplement: Supplementary file 4 [file Image1.PDF]
