# Supplementary material for: To Be or Not To Be T4: Evidence of a Complex Evolutionary Pathway of Head Structure and Assembly in Giant Salmonella Virus SPN3US
Source: Front Microbiol. 2017 Nov 15;8:2251. doi: 10.3389/fmicb.2017.02251 (PMC5694885; doi:10.3389/fmicb.2017.02251)
Supplement: Supplementary file 5 [file Image2.PDF]

Supplementary Figure 2. Peptide coverage of SPN3US vRNAP subunits identified by mass spectrometry.

vRNAP βN

SPN3US_0241 (100%), 159,329.1 Da
TtoT from 213980 to 218194 +
81 exclusive unique peptides, 152 exclusive unique spectra, 203 total spectra, 915/1404 amino acids (65% coverage)

```
GTCMIFLKQF NQRFTVRKTD EFGRPRIIPL EKLQLPRGSI YHTIDLDQVV LAPPLTTPCF GQLEKPAMIR
HHFQLPEDGI IGHPRKKPVV GQERQIFQYH RNNRRIRRMI EDGVVRSDPK VLLIENYTPM LPHYFYPDTL
MSWYERLHNI QLLITNQMKT DSREYMRQNY YIIELGNVLP SFDRFKMTYN DRVKTKLERF KDFNLLWLLE
LFGWAYGDQQ ATLFDGMDMN QLSRVNFIFT HNGGFTSMNM GVIERMRKSA GGRLTDVQMG RMFYRTLTTV
MTVPPSTDYE DDKFVTPEGD ERVHLGEDDD AVDTVANKPE ETLDIVDDEA TQGDNVDDTL NDHEFDEKED
LVIEAPKEDV KPTTIVVERT IPHDAVIKQE VERLAEAGRV TAKAYQFLNE SSKRFVNLPN PYNPKETYGE
GLTIDPKKLE VKAEPKKIER LVTDEAWAEN TTDQIIRQYN QEIFPKDVLG AVASSQRLGL AIHNHTVEKE
ISVTGNIEHH TLRIQPIGGE PTTVRFSIPA LTEDGTWVAN GVEYTMRRQR VDVPIRKVSH DTVALTTAYG
KNFVRRSDKV VNDYGRWLTN AIITRAVDPK NTEITDAKLA NVFDPLSKLP RQYTEVARRV ATFNAIGYQW
NFDIKQKAVF FNEGQLADAD RVELVPVAKK PRSHVIVGMD KNSQLYRVDK DKTEPLGTLA EVFGVDTAKA
PREMTELSLM GKSISLGFVF AYYLGLTGML KHFGISYEVL SVGQRIDKGQ FDSIIRLQDA KIAIVCENDQ
QRMIVNGLDR YIKHFTTYTE SEVDREDIYL NLLRDADGLT PRYIKELKLM RTAFVDDMHA RILRKMGEPE
TFIGLLERSN ELLQTDHTLP EINGDEMMFI GNQRISYHIY TAMVRAMRNY NNAPNSNRKF ELTQDMVWGA
INSDPSVLLA PGANPVQNIK EKDVVTMGGT GGRNRKTMVY STREFQPSDL GIVSGNTVDN GDVGITAFAT
NNPRYDTVDG TTFKKDLDKL KPGEALSFID GLVPDTLMDD AKRQNFVGIQ FGSATSCVGA TTTPYRTEQE
KFVAHRTSVK HARVIDKPGK IVEVLNDHVK IKYDDGEEEA FPLGRWFGPH EGTYYPHTLV TKWKRGDKLP
AGAVITYNEQ HFEPDIYDPT QVSFKNGILA TVALIEGEEV IEDSNAISER FAAKAEADVT KLKEVTMTFT
QNLLEMVKEG DHVDVDSILC TFTDNLTGDM SGFSKEAAET LRELSSFAPR SGVRGFIDKI EVVYHGELED
MTPSLAEMVR KYDRIRRKTA LALKRDEPKD GRVNGDFRVE GVPLAYNSLC VRFYITHRVE MAAADKMVIA
NQLKTTVQEV MYGTNKTEDG QELDIIFGRD SVDARIVGSI MRIGTTNAVG YRGGENIGRI LDGEDVPALP
AKIN
```

vRNAP βC

SPN3US_0218 (100%), 26,858.6 Da
TtoT from 189715 to 190431 +
12 exclusive unique peptides, 24 exclusive unique spectra, 26 total spectra, 92/238 amino acids (39% coverage)

```
RPGTCGSSSA PREDEKMRLD PARRKKAEAY ALQLLNLVDP TGINAEAAKR AFSRMSDEEF NRLREGLPVY
NPTGGKVKID HMRNIKICEA MGLSLCQRLW LTEPKTGILQ RTRYPHLVFR LPARRQTQMQ EKKMAVAKND
KVRDKLSGQV VGPSKASGVS FPEAYIMYSD GHDSTLQEFL WARGGNDTLQ RAFYQSLRQT GKGRINLEGA
ERTSSKAPRT WSSYFKAMHI GNNLGRPE
```

vRNAP β′N

SPN3US_0240 (100%), 62,937.9 Da
TtoT from 212330 to 213970 +
28 exclusive unique peptides, 40 exclusive unique spectra, 41 total spectra, 335/546 amino acids (61% coverage)

```
CFFQFRSLKV RKLNWVDFYL LNVNKSLVSH LKPVTTTDTF QGLTKNFHPE GLYSTEIFGL TGSEARDSTF
SYIDIKLDII SPTVCLALFQ LKQLYEEICS GKRFAIWNEK EKDFEPALPS DKGADTGFNF FLRYYEQLTP
GRNESMRRDE TVDFFNKFRP VSLSRYVLVL PAGLRDLVIR QDGRDQEEEI GGLYRRLISL ARAIPDRSTR
TELTDPVRWK LQQTFNDIWM YFFNIQDGKG GFARRKVTSR KLMNGTRNVL SSFSTGSKVM GREDAIRPTD
TRIGLYQTLK ALLPVAQYHI RERYLSNIRA GDGNLYGVNT KTLKREFLEV SGRVYDLFTT DDGIETLINR
MEARELRHKP LMIDPDHYVA LIYQDKRSFK IFYDIEDLPE GRDRKLVRGL SLAELLYLSG YDIWNDYFSF
ITRYPVTGRG STYSSTIRLE TTTSSLYLHE LEDDWVTLKE KGAISFPDRT VKTFVESMAP HPSRLGGLGG
DYDGDTGSAN CPMSTEALEE NRKWVSSKNY WFATDGSFKI NPVNNVIKRT TAALLK
```

vRNAP β′M

SPN3US_0042 (100%), 49,420.0 Da
TtoT from 39362 to 40660 +
21 exclusive unique peptides, 36 exclusive unique spectra, 54 total spectra, 302/432 amino acids (70% coverage)

```
MMNKIDYLVA ACKAEAWRRL VWRIAVFNVA IFNEKGEPPE QYDLNYIDGL PHYWENEEIK WVPIEGCKKD
EELFVPEEQF ELRPEMYPGL AGPIPTTVGR YVFNWIAIYY AFGTRLPYLA ESRDPLAYRK EMYERCVEYD
DSDPDNEDAI RPYMIGRFVG GLHELAPLCR GIAPTGTIRS LTTHPDAYKV RDALLLKHKD ELDNPAVIVM
IEKALDELDK EWLSGDQSVE FYSSPKARMR RRKLMLMYGI QTAFKEGADF TLIPTSLMEV DQTGMKYLVE
KFNDTREGSF MRGAETAKGG EQVRIIQMIF QNHKIVPGDC GTKLTHALVI NQYNYKRYVG MNAMINGKVT
QLTEEYLKTQ FGKVVRLRRP ILCQQGHVDC CAACASAHKA EEPRAIAADI SSGFSNVMTT AMGAMHGRET
VVKEYIPKFH IT
```

vRNAP β′C

SPN3US_0244 (100%), 27,497.6 Da
TtoT from 220011 to 220745 +
8 exclusive unique peptides, 13 exclusive unique spectra, 15 total spectra, 89/244 amino acids (36% coverage)

```
REVVVSAIET ARRDATKIHA DLVDQGTATI TKGGCYIYIP VGFVAKELAV ISSQVEIVGI FAISTDRKTY
GVSNVTTFIE ITPSAFEEID VQGVPYYEFR FDPGTVVFPN RMLQVLSSPV YNIASYIYDF GNRPFWYTAV
DDAELLSDTK TWNGFTVFND QITADCYAAH TQRKVGDPRT YFRYTLKKDS DLMNRVQFIP LRSGSLNKTS
RLAKIADVEL KQGIRSALQV DPVRAEPLED LYMR
```
