# Supplementary material for: To Be or Not To Be T4: Evidence of a Complex Evolutionary Pathway of Head Structure and Assembly in Giant Salmonella Virus SPN3US
Source: Front Microbiol. 2017 Nov 15;8:2251. doi: 10.3389/fmicb.2017.02251 (PMC5694885; doi:10.3389/fmicb.2017.02251)
Supplement: Supplementary file 6 [file Image3.PDF]

Supplementary Figure 3. Mass spectral peptide coverage of SPN3US head proteins gp243 and gp214.

SPN3US gp243

SPN3US_0243 (100%), 57,070.1 Da
TtoT from 218466 to 220007 +
57 exclusive unique peptides, 126 exclusive unique spectra, 204 total spectra, 402/513 amino acids (78% coverage)

Y G G C A H Y W S H	P G F H Q H Q K R G	S G M I N L Y S L N	A V T N A M K L A K	Q H Q I R L D A D V	D S P V G L L T K A	T T Q Q S V F D T Q
I T D E E F F Q S L	P D I T A L K T P Q	A G T G E A S V I A	D T A A G P E T L N	V E D H E P T L F E	L K R M A I Q R C Q	G M L D F A R N V I
Q P F V Q L V I Q N	N Q G I E N Q A V S	E E W A L V P A G M	D P A L N S P V V Q	A L I D S V E N P L	G N G T T H P A V K	A N V P A D I E L P
E T G S K A Y D D L	V K E L L N A L G W	S I N E A V R T M L	E G N M L S P V S D	R A P H N L K R N I	L F M L L S G Y Y L	E M P W K D S G L N
S G Q W K A Q M L P	L H Y S Y I G W V Y	A Y T Q N I V T R I	K T G N I V F G Y D	N V E K K V Y I C Q	E V M D D Y L E K G	G S V E A L L G A I
Y Q L D E G D T N V	S T R V A S L L E K	Q K D Y I A A W S H	R S A I Q R A K L D	S N W V T R N R Q S	L K Q A F N V A I D	A T D P E L F W R G
E D G Q V A T P E L	I K R S T A S F I D	A F F T T D T T D I	T E F V I K V S A T	E V F G E Y E L S K	L M L D I H R G M I	A N R Q P D E V A T
D W M I N Y V L D W	I C G G V V I S V P	S K V				

SPN3US gp214

SPN3US_0214 (100%), 30,258.0 Da
TtoT from 186782 to 187591 -
21 exclusive unique peptides, 45 exclusive unique spectra, 61 total spectra, 189/269 amino acids (70% coverage)

Y V K D L L C S P I	P Y I F E V D R M N	V N P L A A L S G F	V A K A S T I L P K	A F S G S Q Q E L A	L T T Y D D M S D V	S K W M Q Q E K F L
N F T G M L V P V P	P G F N T Y V M D H	I E R L E S V W A V	L Q K I Q E G V L T	P I D K R F G A M T	H D L G M L T L P I	G F K F K D L N Y P
L K N I N P K D L V	E K L A K S Y T N N	V I D Q R P I E K T	Y H S A G E I D V A	F N R A K A L N L E	V T K K L Q K S I D	R T V E S I N V S V
D I I S N S Q V H P	N V A A E L V K M A	D M G A D W V E L F	G L F M K Q I N E L	T E C L N V T G D R	L K T L K A N K K	
